# Germinal Center B cells provide essential IL-1β signals to TFH cells via canonical NLRP3 inflammasome activity post influenza infection

**DOI:** 10.1371/journal.ppat.1013404

**Published:** 2025-08-18

**Authors:** Juliana Restrepo Munera, Cainan Riccio-Baum, Rebecca Kaddis Maldonado, S. Rameeza Allie

**Affiliations:** Department of Cell and Biological Systems, Penn State College of Medicine, Hershey, Pennsylvania, United States of America; The Ohio State University, UNITED STATES OF AMERICA

## Abstract

Persistent germinal center (GC) responses show increased benefit in optimal responses to influenza infection. Follicular helper T (TFH) cells provide the essential signals and help for maintenance of GCs and require IL-1β signaling for establishment and maintenance. We observe a preferential upregulation of IL-1β within GC B cells and coexpression of NLRP3 and caspase-1 with IL-1β confirms that GC B cells process IL-1β using a canonical NLRP3/caspase-1 mechanism. Using B cell specific ablation of IL-1β production and IL-1β signaling we further confirm that, GC B cells are the primary source of vital IL-1β within the GC and that IL-1β processing by GC B cells post influenza infection is driven by NLRP3 inflammasomes. We observe significant reduction of GC B cells and TFH cells in the absence of B cell derived IL-1β and our analysis of human B cells suggests similar mechanisms in human GC B cells. Our data present GC B cells in two novel roles, the first in producing IL-1β, which is associated with innate functions, within the GC and the second is providing helper cytokine to the TFH cell. Our findings add to the known complexity of the GC providing a target to enhance GC function and persistence.

## Author summary

Follicular helper T (TFH) cells require IL-1B to optimally function within the germinal center (GC). We identify GC B cells as a previously unrecognized source of IL-1β, within the GC microenvironment and demonstrate that B cell–derived IL-1β is necessary for TFH and GC function, including follicular trafficking, even when all other cells retain IL-1β production capacity. We delineate the inflammasome pathway responsible for IL-1β expression in GC B cells and validate these findings in the context of influenza infection and show that analogous pathways operate in the human system, highlighting the broader relevance of B cell–derived IL-1β in shaping adaptive immunity.

## Introduction

Influenza A virus infection poses an annual health risk, primarily in children and elderly populations. In a Global Burden of Disease Study in 2017 it was estimated that influenza-related lower respiratory infection was the cause for 145,000 deaths [1]. Influenza virus is constantly evolving as it has a high mutation frequency, therefore giving the virus a mechanism to evade the host immune response [2]. As a result, we are continuously at risk of influenza infection and therefore we depend on annual vaccination to maintain the humoral response against reinfection. Currently, the vaccine’s efficacy is measured by the production of high affinity antibodies [3], however, the difficulty in predicting the circulating strains of influenza and the virus’s mutation rate leaves us to rely on memory B cells for protection. Following influenza infection, resident memory B cells (BRMs) are established in the lungs, providing local immediate protection against reinfection [4]. Germinal centers (GCs) are microanatomical structures comprising activated B cells that form in secondary lymphoid organs following viral infection or immunization [5], which produce long-lived plasma cells and memory B cells that provide long-term protection against reinfection [6,7]. The establishment of BRMs is dependent on the GC reaction, establishing a need to understand the molecular mechanisms of an optimal GC reaction to provide targets to optimize the protective response against influenza infection [4].

Formation of the GC and its output is dependent on follicular helper T (TFH) cell function, as these cells provide essential signaling molecules to allow for B cell proliferation, survival, selection, maturation and differentiation in the GC reaction, therefore making TFH cells a limiting factor of the GC [8]. TFH cells provide interleukin-21 (IL-21), a cytokine essential for the function of the GC. IL-21, while not required for formation of GC B cells, is required for maintenance of the GC, affinity maturation, and regulation of B cell proliferation in the GC [9–11]. In addition to IL-21, TFH cells express CD40-ligand (CD40L) which signals B cells through the CD40 receptor. CD40 signaling in B cells is vital for GC formation [12,13], aids in B cell trafficking [14], promotes B cell survival [15] and also plays a role in B cell memory development [16]. Apart from IL-21 and CD40 signaling promoting B cell survival, proliferation, and selection in the GC, the two factors synergize to induce c-Myc and p-S6, further stimulating GC B cell selection in the GC [17,18]. In B and T cell interactions, the primary role of B cells is antigen presentation to T cells, however additional roles of B cells, both in the B cell follicle and in the GC, continue to be identified. B cells have been confirmed to be a source of IL-6 that aids in TFH cell function and formation of spontaneous GCs [19,20]. This opens a door to investigate B cells and their contributions in cytokine production to optimize cell-to-cell interactions.

Recent work has shown the requirement of IL-1 signaling by TFH cell for GC regulation. Using Ova-Alum immunization, Ritvo. et al. show that TFH cells express significantly higher IL-1R1, the agonistic receptor for IL-1 signaling, and the requirement for IL-1β for TFH cells to produce IL-21 [21]. It is not known if this requirement for IL-1β by TFH cells is broadly applicable to viral infections. Within the GC, T follicular regulatory cells (TFR) also play a pivotal role in regulating the GC reaction. TFR cells also express IL-1R1, albeit at significantly lower levels than TFH cells, while also expressing IL-1 decoy receptor IL-1R2 and antagonist receptor IL-1Ra. Deletion of IL-1R1 on TFR cells does not affect GC B cell numbers, therefore suggesting that IL-1R1 on TFH cells, but not TFR cells, is essential for maintaining the GC [22]. Deletion of IL-1R2 on TFR cells, which in turn increases the availability of IL-1 signaling, does result in a heightened GC and TFH cells numbers in a mouse model with sheep red blood cells immunization [23]. In addition to highlighting the importance of IL-1 signaling for the GC reaction, this data supports the need for a continuous supply of IL-1β within the GC to promote the TFH function. If IL-1β is vital for TFH cell function and maintenance in the GC, it may suggest that IL-1β is the limiting factor for the GC reaction.

As TFH cells come from the T cell zone they can receive IL-1β from dendritic cells in the T cell zone [24]. However, as cytokine signaling occurs in the immediate vicinity of release, this may not be sufficient as TFH cells cross the T-B border and establish residence in the GC. If TFH cells need an ongoing source of IL-1β within the follicle, especially within the GC, it is important to identify the cell type that provides this localized IL-1β in the GC. As the largest population of cells in the GC are B cells, we hypothesized that GC B cells are the vital source of IL-1β for IL-1signaling in TFH cells. Here we show that in comparison to naïve B cells or activated B cells which are not in the GC, GC B cells exhibit the highest expression of IL-1β. Using B cell specific ablation of IL-1β production and T cell specific ablation of IL-1R1 signaling post influenza infection, we show that B cell-derived IL-1β is essential for the establishment of TFH cells and therefore GCs. We further determine the cellular mechanisms that induce the production of IL-1β in GC B cells and we observe that these mechanisms are analogous in human GCs.

## Results

### TFH responses in influenza infection depend on IL-1 receptor signaling

**Fig 1:**
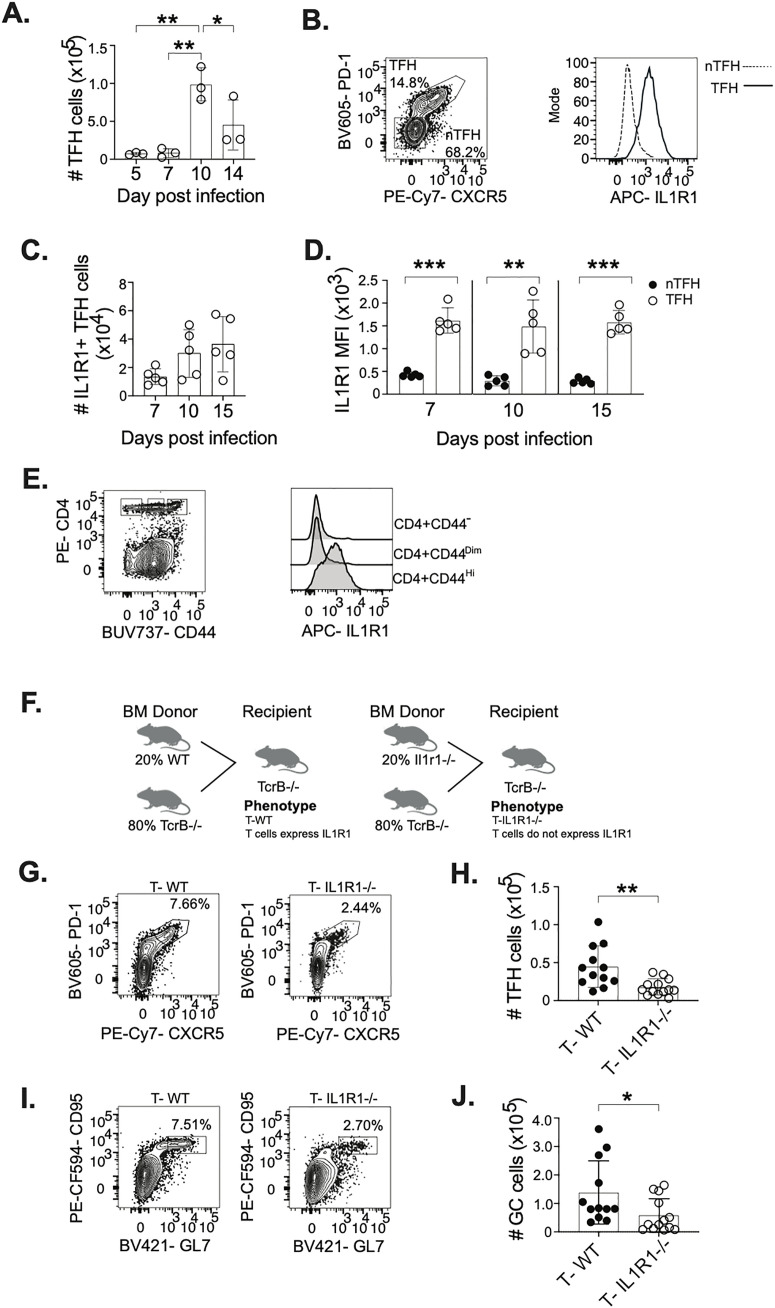
Preferential expression of IL-1R1 on TFH cells following influenza infection. Mediastinal lymph nodes (mLN) from A/PR8 infected C57BL/6J mice were gated on live, singlet lymphocytes (**S1****A**). (**A**) TFH cell (CXCR5 + PD-1+) numbers were gated from CD4 + CD19-CD25- (S1A Fig) at 5, 7, 10, and 14 days post infection (dpi). (**B**) IL1R1 expression (S1D and S1E Fig) was examined on TFH (CXCR5 + PD-1+) and non-TFH (nTFH) (CXCR5-PD-1-) cells and used to quantify (**C**) number of IL1R1 + TFH cells at 7, 10, and 15 dpi. (**D**) IL1R1 was quantified by mean fluorescence intensity (MFI) in TFH and nTFH cells at 7, 10, and 15 dpi. (**E**) IL1R1 expression was compared between CD4 + T cells expressing different levels of CD44. (**F**) Schematic shows the formation of 80:20 ratio T cell specific bone marrow chimeras. 80:20::Tcrβ^-^:IL1R1^-/-^ and 80:20::Tcβ^-^:WT to generate a T cell specific IL1R1 knockout mouse (T-IL1R1^-/-^) and a WT control (T-WT), respectively (Created in BioRender. Restrepo, J. (2025) https://BioRender.com/0r4q7ep). At 10 dpi, mLN from A/PR8 infected T-IL1R1^-/-^ and T-WT mice were gated on live, singlet lymphocytes (S2A Fig) (**G**) TFH cells (CXCR5 + PD-1+) were gated on CD4 + CD19-CD25- (S2B Fig) and (**H**) quantified TFH cell number. (**I**) GC B cells were gated on CD19 + cells (S2B Fig) to (**J**) quantify GC B cell numbers. Data is representative of 3 experiments with 3 mice (**A**), 2 experiments with 5 mice per timepoint (**B-E**) and 3 experiments with graphs showing individual points and mean±SD (**G-J**). *p < 0.05, **p < 0.01, ***p < 0.001, and ****p < 0.0001.

IL-1β signaling occurs via the primary IL-1 Receptor 1 (IL-1R1) complexing with the accessory chain IL-1R3 [25,26]. Dendritic cell derived IL-1β activity on T cells led to numerous studies showing the direct impact of IL-1β on T cell function via IL-1R1 signaling for over two decades [27]. Studies on protective responses to influenza infection, which are predicated on an optimal B cell response, show that sustained GCs are vital to this outcome [28]. As TFH cells are the limiting factor of the GC response [29], and TFH cells require IL-1β post immunization for their function [21], we investigated the role of IL-1 signaling in the GC response following influenza A infection. Kinetics of TFH cells (CD19-CD4 + CD25-CXCR5 + PD-1+) (S1A Fig) following influenza (A/PR8) infection show that TFH cells peak at 10 days post infection (dpi) (Fig 1A). To begin, IL-1R1 expression was examined using a combination of a primary and secondary antibodies, with a modified fluorescence minus one (FMO) control lacking the primary antibody (secondary only control) (S1B Fig). We determined that TFH cells expressed IL-1R1 at 10dpi and compared it to non-TFH (nTFH) cells (Fig 1B). The number of IL-1R1 expressing TFH cells increase between 7 and 10 dpi (Fig 1C), with TFH cells expressing significantly higher quantities of IL-1R1 compared to nTFH cells in the mediastinal lymph node (mLN) at days 7, 10, and 15 post infection (Fig 1D). We examined nTFH and TFH cells for CD44 expression, as a marker of antigen experience [30], with TFH cells primarily found in the CD4 + CD44^Hi^ population, while nTFH cells make up the majority of both CD4 + CD44^Dim^ and CD4 + CD44- T cells. Further, we compared IL-1R1 expression in CD4 + CD44-, CD4 + CD44^Dim^, and CD4 + CD4^Hi^, observing that CD4 + CD44^Hi^ express significantly higher IL-1R1, while CD4 + CD44- and CD4 + CD44^Dim^ lack IL-1R1 expression (Fig 1E), therefore showing that antigen exposure drives upregulation of IL-1R1 on T cells.

Preferential increase in IL-1R1 expression on TFH cells suggests a functional relevance in the GC, but we wanted to confirm that T cell specific IL-1R1 signaling had functional consequences within the GC post influenza infection. To determine the requirement of IL-1 signaling on T cells for the formation or maintenance of the GC, we generated T cell specific wildtype (WT) or IL-1R1 knockout (KO or -/-) mice. We made 80:20 mixed bone marrow chimeric mice. T-WT mice were made with 20% C57BL/6 bone marrow and 80% T cell deficient bone marrow. Therefore, T cells and all other cells in these mice are WT. T-IL1R1^-/-^ mice were made with 20% C57BL/6 IL-1R1^-/-^ bone marrow and 80% T cell deficient bone marrow. Therefore, all T cells and 20% of other cells in these mice are IL-1R1^-/-^ but most of the other cell types in these mice are WT. (Fig 1F). At 10dpi with A/PR8, flow cytometry analysis of the mLN of T-WT and T-IL1R1^-/-^ mice (S2A and S2B Fig) showed a significant reduction in TFH (Fig 1G) in which we observed significantly lower TFH cell numbers in T-IL1R1^-/-^ mice compared to T-WT mice (Fig 1H). Consequentially, GC B cell (Fig 1I) numbers were significantly lower in T-IL1R1^-/-^ mice than in T-WT mice (Fig 1J). These findings confirm the significance of IL-1 signaling on TFH cells on GC function, post influenza infection. This raises the question about the source of IL-1β within the follicle and especially within the GC.

### B cells responding to T helper-like stimulation and GC B cells post infection express IL-1β

**Fig 2:**
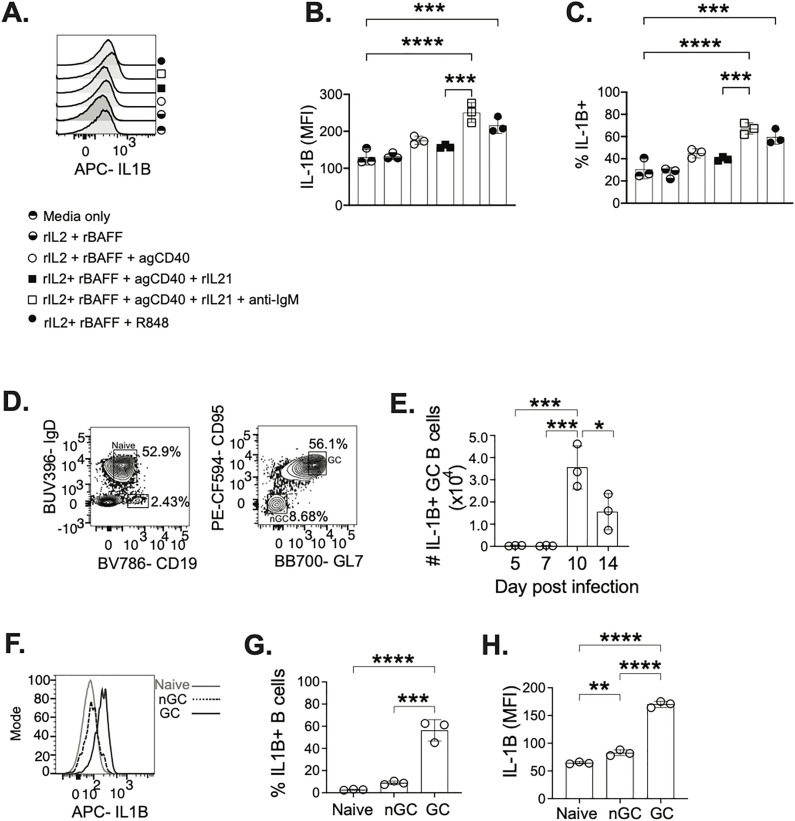
GC B cells express IL-1β after influenza infection. Splenic B cells were enriched from naïve or A/PR8 infected mice at 15 dpi and stimulated with a combination of rBAFF, rIL2, R848, anti-CD40, rIL21, anti-IgM to examine intracellular (IC) IL-1β. Cells were gated on live, singlet lymphocytes (S3A Fig) to examine (**A**) B cells expressing IC IL-1β in each stimulation group. IL-1β expression in B cells was quantified by (**B**) MFI and by (**C**) frequency of IL-1β + B cells. (**A-C,** S3B Fig) Symbols representing each stimulation match their respective histogram and bar on the graphs. mLN from A/PR8 infected C57BL/6J mice were gated on live, singlet lymphocytes (S4A Fig) to determine (**D**) B cell populations including naïve, GC, and non-GC (nGC) B cells. (**E**) B cell populations were examined for IC IL-1β at 5,7,10,14 days post infection. (**F**) IC IL-1β was compared between naïve, GC and nGC B cells to determine the (**G**) frequency of IL-1β+ cells in each respective population and (**H**) quantify the MFI of IL-1β in each respective B cell population (S4B and S4C Fig). Data are representative of 3 experiments with technical triplicates per stimulation group (**A-C**) and 3 experiments with 3 mice and graphs show individual points and mean±SD (**C-G**). *p < 0.05, **p < 0.01, ***p < 0.001, and ****p < 0.0001.

In the T cell zone, T cells have access to DCs as a source of IL-1β, but within the GC the largest and closest population to the TFH cell is the GC B cell. Therefore, we investigated GC B cells as the source of IL-1β. As there is considerable crosstalk between TFH cells and GC B cells, we examined how T cell help could stimulate B cells to express IL-1β. Isolated splenic B cells from A/PR8 infected C57BL/6 mice at 15dpi (S3A Fig) were stimulated with factors mimicking CD4 T cell help (CD40 and IL-21) and B cell receptor (BCR) stimulation (anti-IgM) [31] (Fig 2A). IL-1β expression was measured on a per cell basis (MFI), frequency of B cells expressing IL-1β, and number of IL-1β + B cells (Figs 2B, 2C and S3B). Stimulation with CD40 and IL-21 of B cells showed an increase in IL-1β, however the highest upregulation in IL-1β expression was observed when B cells were stimulated with CD40, IL-21, and anti-IgM (Fig 2A–2C). Therefore, we show that both B cell receptor stimulation and T cell help are essential for optimal IL-1β production by B cells. Additionally, B cells isolated from either naïve C57BL/6 mice (S3C Fig) and from A/PR8 infected mice (S3D Fig) were compared for IL-1β expression. Interestingly, we observed that CD40 + IL-21 stimulation of B cells from A/PR8 infected mice have a 3-fold higher expression of IL-1β than stimulated B cells from naïve mice (S3C Fig compared to S3D Fig). This suggests that the GC response to influenza infection promotes B cells expression of IL-1β required for the crosstalk between B and T cells post infection.

To further examine B cells as a source of IL-1β *in vivo*, we examined naïve B cells (CD19 + IgD+), activated but non-GC (nGC) B cells (CD19 + IgD-GL7-CD95-), and GC B cells (CD19 + IgD-GL7 + CD95+) post A/PR8 infection (Figs 2D and S4A–S4C). As experimental controls for IL-1β staining we used FMOs respective to all three cell types, but we used the combination of the visible positive population on the nGC cells and the FMO on the GC B cell to place our gate and avoid false positive staining (S4C Fig). IL-1β expressing GC B cells peak at 10 days post influenza infection (Fig 2E), coinciding with the kinetics of TFH cells (Fig 1A) also peaking at day 10. This supports the hypothesis that the production of IL-1β by GC B cells fuels the TFH cells to peak at 10 dpi in the GC. Additionally, intracellular IL-1β expression was examined in naïve, nGC, and GC B cells (Fig 2F). We observed that a significantly higher frequency of GC B cells expressed intracellular IL-1β, compared to the frequency of nGC and naïve B cells expressing IL-1β (Fig 2G). On a per cell comparison, we also observe GC B cells expressing significantly more IL-1β than nGC and naïve B cells (Fig 2H). As an alternative analysis, we also examined IL-1β in GL7^Hi^, GL7^Dim^, and GL7- B cells observing highest IL-1β expression in GL7^Hi^ B cells, a population composed primarily of GC B cells (S4D and S4E Fig). Among the cells that interact with TFH cells, GC B cells may be the prime candidate as a source of IL-1β for the TFH cell. Yet, to rule out other residents of the GC, we examined follicular dendritic cells (FDCs) which reside in the GC. GC B cells express much higher levels of IL-1β compared to FDCs (S5C Fig). Additionally, as FDCs have limited to no direct interactions with TFH cells, they may not be the likely source of IL-1β for TFH IL-1 receptor signaling.

### IL-1β coexpression with components of canonical inflammasome activity

**Fig 3:**
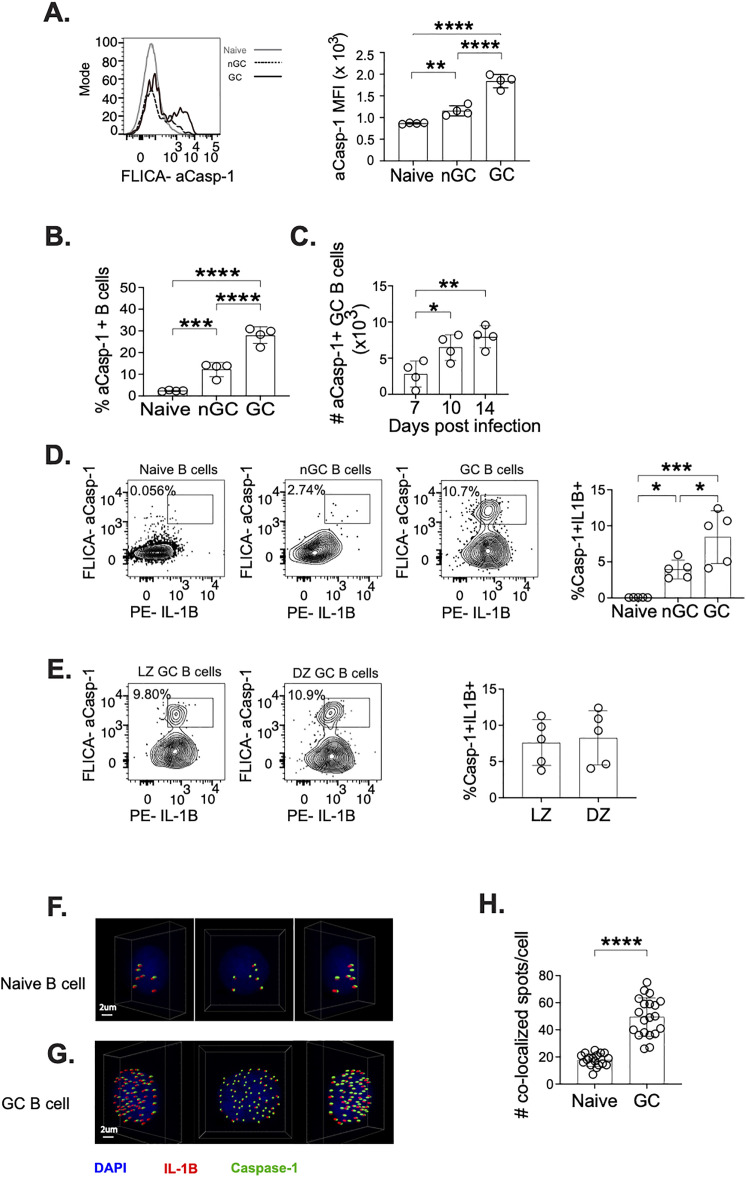
GC B cells display higher expression of active caspase-1 and preferential coexpression of caspase-1 and IL-1β. mLN from A/PR8 infected C57BL/6J mice at 10 dpi were gated on live, singlet lymphocytes (S5A Fig). Gating from naïve, GC, and nGC (S5B Fig) was used to determine caspase-1 and IL-1β expression. (**A**) Expression of active caspase-1 was determined by flow cytometry using a fluorochrome-labeled inhibitor of caspase and the MFI was used to quantify active caspase-1 in respective populations. (**B**) Frequency of active caspase-1 at 10 dpi was used to compare active caspase-1 expression in naïve, nGC, and GC B cells. (**C**) The number of active caspase-1 expressing GC B cells were determined at 7,10, and 15 dpi. At 10dpi, (**D**) coexpression of IC IL-1β and active caspase-1 was determined in naïve, GC, nGC B cells. Additionally, (**E**) IL-1β and active caspase-1 coexpression was compared between light zone and dark zone GC B cells (S5D Fig). Pooled mLN from 20 A/PR8 mice at 10dpi were sorted for live naïve (CD19 + IgD+) (S6A Fig) and GC (CD19 + GL7+) (S6B Fig) B cells. Naïve and GC B cells were cytospun and stained for IL-1β (red), caspase-1 (green) and dapi (blue). Confocal microscopy along with spot colocalization analysis was used to determine colocalization (spots) of IL-1β and Caspase-1 in (**F**) naïve and (**G**) GC B cells. (**H**) Co-localization spots were quantified in naïve and GC B cells (S6C and S6D Fig). (**A-C**) Data are representative of 2 experiments with 4 mice at each time point, (**D-E**) is representative of 2 experiments with 4 mice examined at 10dpi, and (**F-H**) are representative of 1 experiment with 20 total lymph nodes pooled and 20 cells per group imaged for spot co-localization quantification. Graphs show individual points and mean±SD. *p < 0.05, **p < 0.01, ***p < 0.001, and ****p < 0.0001.

IL-1β is produced as an immature peptide, pro-IL1β, that must be cleaved to form the mature and active IL-1β, that can then be secreted [32]. The enzyme most often related to activation of IL-1β is caspase-1. Caspase-1 also exists in an inactive form, pro-caspase-1, and once activated can cleave pro-IL-1β into IL-1β [33]. We therefore examined intracellular expression of active caspase-1. This was done using a fluorochrome-labeled inhibitor of caspase (FLICA), which covalently binds to the active form of caspase-1, allowing us to specifically identify cells expressing active caspase-1 [34]. Expression of active caspase-1 was significantly higher in GC B cells in comparison to naïve and nGC B cells (Figs 3A, S5A and S5B). Additionally, a higher percentage of GC B cells express active caspase-1 than the percentage of nGC and naïve B cells expressing caspase-1 (Fig 3B). To determine if the expression pattern of active caspase-1 followed the expression of IL-1β in GC B cells (Fig 2D) we determined active caspase-1 at 7-, 10- and 15-days post A/PR8 infection, observing the number of GC B cells expressing active caspase-1 peak at day 10 (Fig 3C). This increase of active caspase-1 at 10dpi along with the peaking of IL-1β at 10dpi (Fig 2E), which in turn coincides with the peak of TFH cells (Fig 1A) at 10dpi, points to 10dpi as a consequential timepoint to examine the impact of IL-1β on GC function at 10dpi.

As we observed both IL-1β expression and active caspase-1 in GC B cells, we wanted to further determine if the GC B cells were simultaneously expressing both IL-1β and active caspase-1. The coexpression of IL-1β and active caspase-1 would serve as a correlate of active IL-1β production. We observe that GC B cells have a significantly higher frequency of cells exhibiting coexpression of caspase-1 and IL-1β in comparison to nGC and naïve (Fig 3D). We also examined IL-1β coexpression in GC B cells in the light zone (LZ) and dark zone (DZ) of the GC (S5F Fig), however we did not observe significant difference in coexpression between LZ and DZ GC B cells (Fig 3E). At 10dpi, the GC is in its early stages and as it is still small in size the production of IL-1β may not be regulated between the zones. To further confirm that caspase-1 is interacting with pro-IL-1β to execute cleavage into IL-1β, we sorted naïve or GC B cells from the mLN of A/PR8 infected mice (S6A and S6B Fig) and used confocal microscopy to determine co-localization of caspase-1 and IL-1β in single naïve and GC B cells (Figs 3F, 3G, S6C and S6D). Co-localized spots were identified when caspase-1 and IL-1β were 4μm or less from each other, with such proximity indicating protein interactions, and therefore IL-1β activation. We observed significantly more spots of colocalized caspase-1 and IL-1β in GC B cells than in naïve B cells (Fig 3H), displaying interaction between caspase-1 and IL-1β, as well as suggesting inflammasome activity.

**Fig 4:**
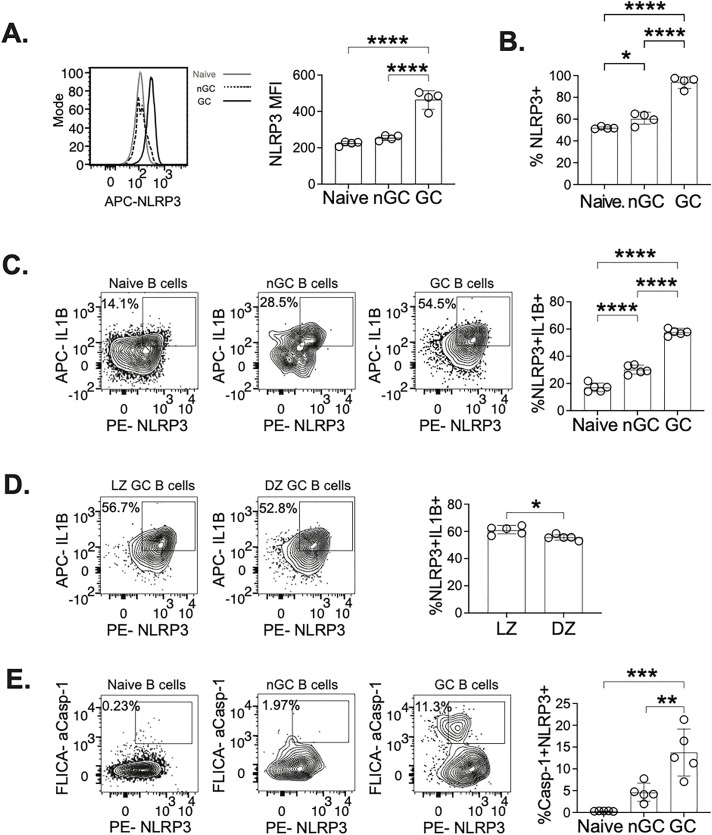
GC B cells exhibit enhanced coexpression of NLRP3 with caspase-1 and IL-1β. mLN from A/PR8 infected C57BL/6J mice at 10 dpi were gated on live, singlet lymphocytes (S7A Fig) followed by a comparison of naïve, nGC, and GC B cell populations (S7B Fig). (**A**) Mean fluorescent intensity (MFI) of intracellular NLRP3, examined by flow cytometry to show per cell expression. (**B**) Proportion of cells expressing NLRP3. Comparing GC B cells to non-GC and naïve B cells. (**C**) Coexpression of IL-1β and NLRP3 examined in GC, nGC, and naïve B cells and frequency of IL-1β+NLRP3 + cells were quantified. (D) coexpression of IL-1β and NLRP3 was further examined in light zone and dark zone GC B cells. Additionally, (**E**) Frequency of cells with coexpression of active caspase-1 and NLRP3 was measured in GC, nGC, and naïve B cells. (**A and B**) data representative of 2 experiments with 4 mice at 10dpi, and (**C-E**) data representative of 2 experiments with 5 mice at 10dpi. Graphs show individual points and mean±SD. *p < 0.05, **p < 0.01, ***p < 0.001, and ****p < 0.0001.

The mechanism of caspase-1 activation necessitates the formation of a multimolecular complex, an inflammasome, which requires a sensor protein, an adaptor protein, and pro-caspase-1 to oligomerize. Oligomerization of these proteins then allows pro-caspase-1 to undergo autocleavage, in which active caspase-1 can then cleave pro-IL1β [35]. The most commonly studied inflammasome is the NLRP3 inflammasome, in which its sensor protein is NOD-like receptor protein 3 (NLRP3), and its adaptor protein is Apoptosis-associated speck-like protein containing a caspase recruitment domain (ASC). NLRP3, unlike most other inflammasomes, responds to a variety of stimuli, including changes in intracellular ion flux, reactive oxygen species (ROS), and extracellular ATP [36]. GC B cells are not being directly infected by A/PR8, however they undergo rapid cycles or proliferation and selection leading to high quantities of cell death within the GC causing a hypoxic environment with high concentrations of extracellular ATP [37–39]. As GC B cells are susceptible to environmental stimuli, we hypothesized that IL-1β production by B cells would be mediated by the NLRP3 inflammasome. We examined intracellular NLRP3 expression in naïve, nGC, and GC B cells at 10 dpi (Figs 4A, S7A and S7B). We observed significantly higher NLRP3 on a per cell basis in GC B cells compared to naïve and nGC B cells (Fig 4A), along with high percentage of GC B cells expressing NLRP3 compared to the percentage of naïve and GC B cells (Fig 4B). To determine if NLRP3 is likely to be involved in IL-1β production, we examined coexpression of IL-1β and NLRP3. We found that the frequency of GC B cells coexpressing IL-1β and NLRP3 is significantly higher in GC B cells (Figs 4C, S7C and S7D). Additionally, we also compared IL-1β and NLRP3 coexpression in LZ and DZ GC B cells. We observed a statistically significant increase in coexpression of IL-1β+NLRP3+ in LZ GC cells (Fig 4D), but we recognize that the difference is small. To further support our hypothesis of NLRP3 inflammasome activity in GC B cells, we also examined coexpression of active caspase-1 and NLRP3. Similar to our previous observations of GC B cells having higher frequencies of IL-1β and active caspase-1 coexpression (Fig 3D), we also observe that GC B cells have significantly higher percentage of cells coexpressing NLRP3 and active caspase-1 (Fig 4E) than nGC and naïve B cells. Collectively, these observations support the intracellular mechanism by which B cells produce IL-1β is the NLRP3 inflammasome, with GC B cells being the predominant B cell population involved in IL-1β production.

### Germinal center response post influenza infection is dependent on B cell derived IL-1β

**Fig 5:**
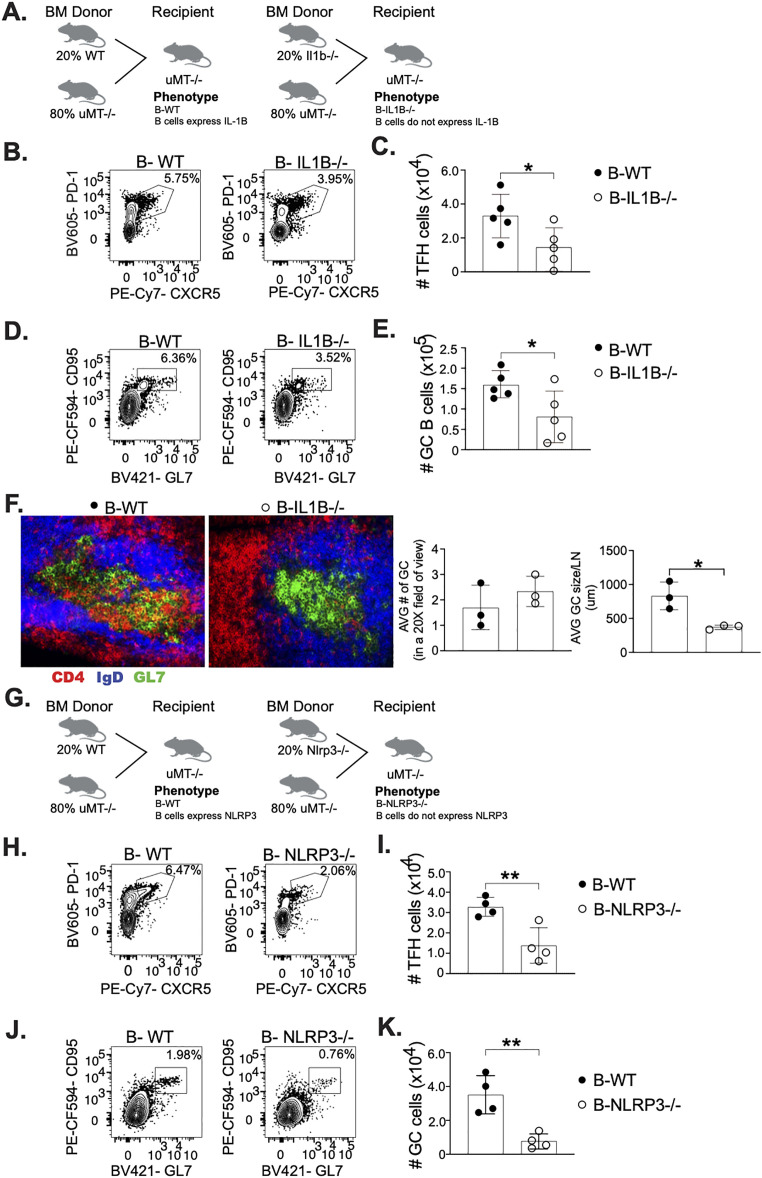
B cell derived IL-1β and NLRP3 are required for optimal GCs following influenza infection. (**A**) Schematic of B cell specific IL-1β^-/-^ bone marrow chimeras. muMT recipients received donor bone marrow cells at 80:20 ratio of muMT^-^:IL1β^-/-^ and 80:20 ratio of muMT^-^: WT to generate a B cell specific IL-1β knockout mouse (B-IL1β^-/-^) and a WT control (B-WT), respectively (Created in BioRender. Restrepo, J. (2025) https://BioRender.com/0r4q7ep). At 10 dpi, mLN from A/PR8 infected B-WT and B-IL1β^-/-^ mice were gated on live, singlet lymphocytes (S8A Fig). (**B**) TFH cells were gated on CD4 + CD19-CD25- cells (S8B Fig) to (**C**) quantify TFH cell numbers. (**D**) GC B cells were gated on CD19 + cells (S8B Fig) to (**E**) quantify GC B cell numbers. Immunofluorescent imaging was used to examine GC B cells (GL7, green), naïve B cells (IgD, blue) marking the B cell follicle, and helper T cells (CD4, red) (**F**) to quantify GC numbers in a 20x field of view as well as measure size of GCs, along with examining CD4 + T cell infiltration into the B cell follicle. (**G**) Schematic of B cell specific NLRP3^-/-^ bone marrow chimeras. muMT recipients received donor bone marrow cells at a 80:20 ratio of muMT^-^:NLRP3^-/-^ and muMT^-^: WT to generate a B cell specific NLRP3 knockout mouse (B-NLRP3^-/-^) and a WT control (B-WT), respectively (Created in BioRender. Restrepo, J. (2025) https://BioRender.com/0r4q7ep). At 10 dpi, mLN from A/PR8 infected 80:20 B-NLRP3^-/-^ and B-WT mice were gated on live, singlet lymphocytes (S8C Fig). (**H**) TFH cells were gated on CD4 + CD19-CD25- cells (S8D Fig) to (**I**) quantify TFH cell numbers. (**J**) GC B cells were gated on CD19 + cells (S8D Fig) to (**K**) quantify GC B cell numbers. (**B-E**) Data are representative of 2 experiments with 4-5 mice per groups. (**F**) Data are representative with graphs showing average GC number and/or size in from 3 different mLN. (**H-K**) data are representative of 2 experiments with 4-5 mice per groups. *p < 0.05.

Our observation of IL-1β production by B cells lead us to investigate if B cell intrinsic IL-1β and NLRP3 significantly impacted GCs post influenza infection. First, we generated a B cell specific IL-1β KO bone marrow chimeric mouse. Similar to Fig 1F, 80% of the bone marrow cells from B cell deficient muMT mice are combined with 20% bone marrow cells from either C57BL/6 WT or C57BL/6 IL-1β^-/-^ mice and transferred to lethally irradiated muMT recipient mice. B cell specific IL-1β KO mice (B-IL1B^-/-^) will have all B cells deficient in IL-1β while all other cells are WT. WT control mice (B-WT) will have all cells, including B cells, expressing IL-1β (Fig 5A). At 10 dpi, lymphocyte populations were examined in B-IL1B^-/-^ and B-WT mice (S8A and S8B Fig). TFH cells (Fig 5B) were quantified, and they had significantly lower cell numbers in B-IL1β^-/-^ mice compared to B-WT mice (Fig 5C), suggesting that B cell-derived IL-1β is important for the establishment of TFH cells. We examined GC B cells (Fig 5D) and similarly observed significant decreases of GC B cell numbers in the absence of B cell IL-1β (Fig 5E), as well. Immunofluorescent imaging (IF) was also used to examine GCs, at a 20X field of view, and compare GC quantity and size in B-IL1β^-/-^ and B-WT mice. While no significant differences were observed in the frequency of GCs in a field of view, GCs in B-WT mice were significantly larger in size than GCs in in B-IL1β^-/-^ (Fig 5F). The large GC size observed by IF complements the data in Fig 5D and 5E showing increased GC B cell numbers in B-WT mice. Importantly, post influenza infection B-IL1β^-/-^ also have noticeably less infiltration of CD4 + T cell into the GC and even the follicular areas, compared to B-WT mice (Figs 5F and S8C). These findings demonstrate the requirement for B cell derived IL-1β for optimal GC function by the establishment of TFH and GC B cells post influenza infection.

As we observed NLRP3 inflammasome activity along with IL-1β production in B cells, we also explored the impact of B cell derived NLRP3 on the establishment of GCs (S8D and S8E Fig). As previously explained in Fig 5A, we generated a B cell specific NLRP3 KO bone marrow chimera (B-NLRP3^-/-^) and a WT control (B-WT) (Fig 5G). As observed in B cell specific IL-1β KO mice, a B cell specific depletion of NLRP3 also results in significant decreases in TFH cells numbers (Fig 5H and 5I) as well as in GC B cell numbers (Fig 5J and 5K). The requirement for B cell derived NLRP3, post influenza infection, further supports NLRP3 activity as a mechanism of IL-1β production in B cells.

### AIM2 inflammasome does not significantly impact germinal center B cells post influenza infection

**Fig 6:**
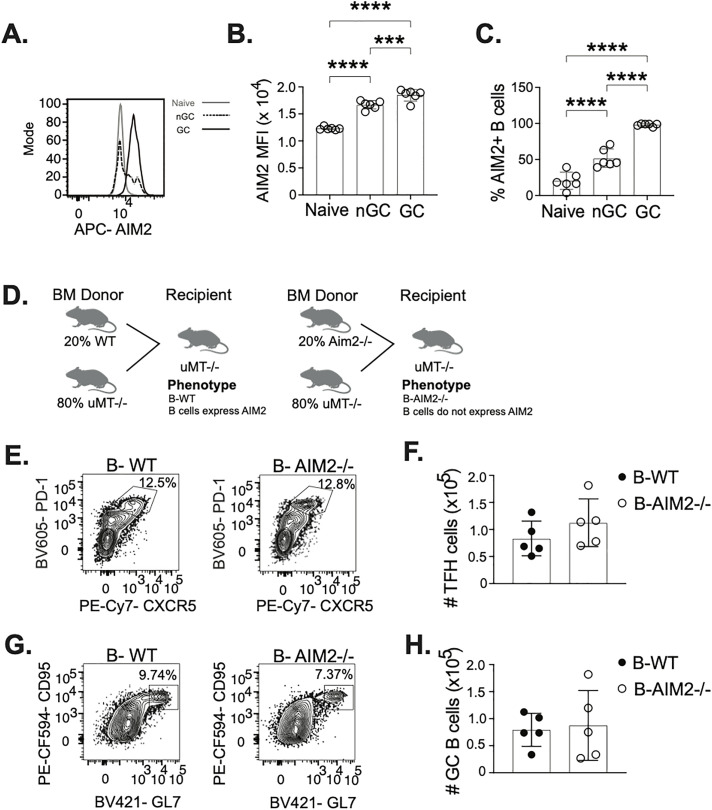
AIM2 inflammasome activity is not essential for the establishment of GCs. mLN from day 10 A/PR8 infected C57BL/6J mice were gated on live, singlet lymphocytes. (**A**) Gating from naïve, GC, and nGC was used to determine AIM2 expression (S9A Fig). (**B**) AIM2 MFI was quantified as well as (**C**) frequency of AIM2 + cells in the B cell subsets. (**D**) Schematic of B cell specific AIM2^-/-^ bone marrow chimeras (Created in BioRender. Restrepo, J. (2025) https://BioRender.com/0r4q7ep). muMT recipients received donor bone marrow cells at a 80:20 ratio of muMT^-^:AIM2^-/-^ and muMT^-^:WT to generate a B cell specific AIM2 knockout mouse (B-AIM2^-/-^) and a WT control (B-WT), respectively. At 10 dpi, mLN from A/PR8 infected B-AIM2^-/-^ and B-WT mice were gated on live, singlet lymphocytes (S9B Fig). (**E**) TFH cells were gated on CD4 + CD19-CD25- cells (S9C Fig) to (**F**) quantify TFH cell numbers. (**G**) GC B cells were gated on CD19 + cells (S9C Fig) to (**H**) quantify GC B cell numbers. (**A-C**) data representative of 2 experiments with 5 mice at 10dpi, and (**F and H**) data representative of 3 experiments with 5 mice at 10dpi. *p < 0.05, **p < 0.01, ***p < 0.001, and ****p < 0.0001.

IL-1β production can employ various mechanisms and NLRP3 mediated processing of IL-1β is one of many. Therefore, it is important for us to rule out other inflammasome activities as being significant for IL-1β driven GC function post influenza infection. Most inflammasomes are triggered by bacterial components as they are lipopolysaccharide (LPS) sensors. In mice, only 2 main inflammasomes, AIM2 and NLRP3, respond to viral infection. Absent in melanoma 2 (AIM2) is a sensor protein that responds to double stranded DNA (dsDNA) in the cytoplasm, making it a sensor of DNA viruses. While AIM2 can be triggered by DNA viruses, it can also be triggered by host cytoplasmic DNA [40]. Considering the potential of DNA damage and mitochondrial damage resulting from the continuous B cell turnover in the GC, we decided to explore the role of the AIM2 inflammasome. In response to cytoplasmic dsDNA, AIM2 oligomerizes with ASC and caspase-1, similar to NLRP3, to form an inflammasome complex [41]. We first examined intracellular AIM2 expression (S9A Fig) in naïve, nGC, and GC B cells (Fig 6A). We observed significantly more AIM2 intracellular expression in GC B cells than in nGC and naïve B cells (Fig 6B), along with higher frequency of AIM2 expressing GC B cells than nGC and naïve B cells (Fig 6C). As we observe the majority of GC B cells expressing AIM2, we sought to investigate if AIM2 has a significant functional role on the GC, following influenza infection. We generated a B cell specific AIM2 KO bone marrow chimera (B-AIM2^-/-^) (Fig 6D), as explained in Fig 5A, along with a WT control (B-WT). At 10 days post A/PR8 infection, GC cell populations were examined (S9B and S9C Fig). We did not observe significant differences in TFH cell numbers between B-AIM2^-/-^ and B-WT mice (Fig 6E and 6F). Additionally, there were no significant differences in GC B cell numbers between B-AIM2^-/-^ and B-WT mice (Fig 6G and 6H). These findings further support our hypothesis that GC B cells produced IL-1β in an NLRP3 dependent manner, and other inflammasomes are unlikely to be involved in GC B cell derived IL-1β after influenza infection.

### Human germinal center B cells mimic mechanisms of IL-1β production observed by mouse GC B cells

**Fig 7:**
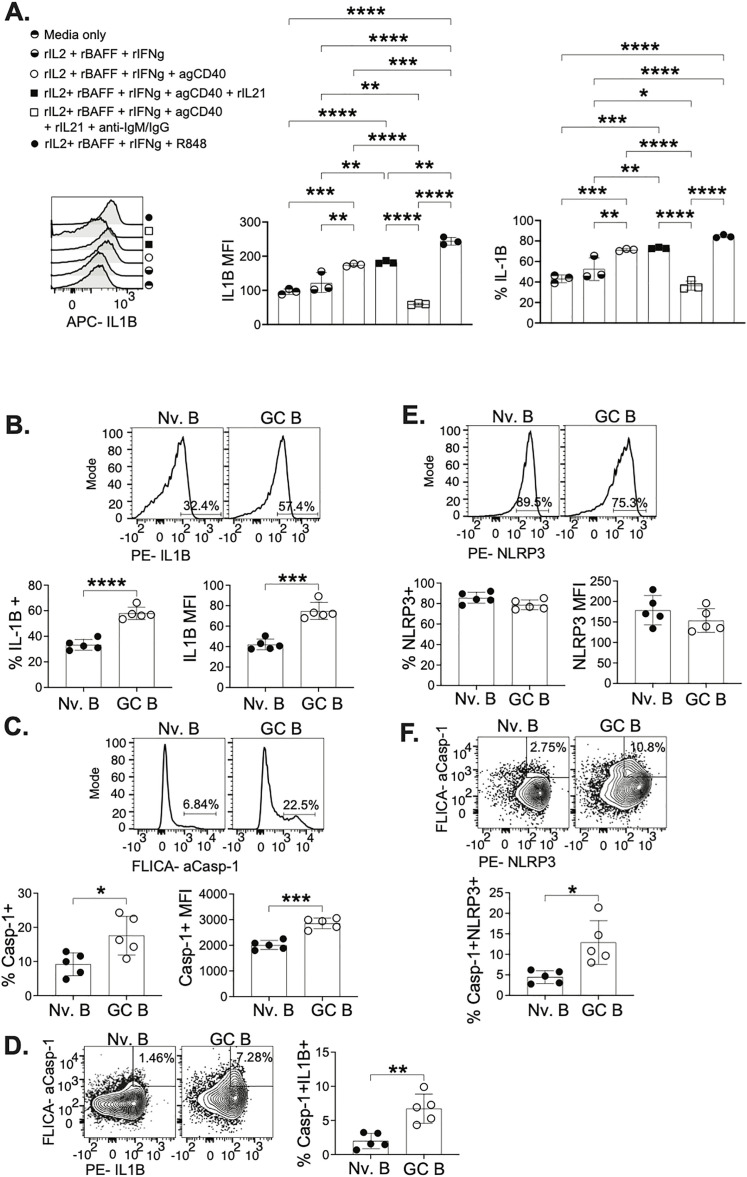
Human GC B cells show preferential expression of IL-1β, active caspase-1, and their coexpression. B cells enriched from human tonsils were stimulated with a combination of rBAFF, rIL2, rIFNg, R848, anti-CD40, rIL21, anti-IgM, and anti-IgG to examine IC IL-1β. Cells were gated on live, singlet lymphocytes (S10A Fig) to examine B cells expressing IC IL-1β (S10B Fig). (**A**) IL-1β was quantified by MFI and frequency f IL-1β + B cells. Symbols representing each stimulation match their respective histogram and bar on the graphs. Human tonsils were gated on live, single lymphocytes (S10C Fig) to examine naïve (CD19 + CD3-CD38-IgD+) and GC/active (CD19 + CD3-CD38 + IgD-) (S10D Fig) and intracellular IL-1β, caspase, and NLRP3 (S10D Fig). Naïve and GC B cells, from human palatine tonsils, were compared for (**B**) IL-1β expression, (**C**) active caspase-1, and (**D**) coexpression of IL-1β and active caspase-1. Tonsil naïve and GC B cells were examined for (**E**) intracellular NLRP3 expression and (**F**) coexpression of NLRP3 and active caspase-1. (**A**) Data are representative of 4 experiments with technical triplicates per stimulation group. (**B-F**) Data are representative of 2 experiments with 4-5 patient samples per experiment. *p < 0.05, **p < 0.01, ***p < 0.001, and ****p < 0.0001.

Recent findings post influenza vaccination in humans uncovers the benefits of sustained GCs for the formation of matured antibody responses to influenza [42]. As our data shows that IL-1β production by B cells in influenza infected mice is necessary for optimal establishment of GCs, we wanted to determine if similar mechanisms are at play in human GC B cells. First, we examined if human B cells stimulated with factors mimicking TFH cells help (CD40 and IL-21) could produce IL-1β. We isolated B cells from palatine tonsils (S10A and S10B Fig) and stimulated with survival factors (IL-2 + BAFF+IFNg), TFH help factors (CD40 and IL21), B cell receptor stimuli (anti-IgM and anti-IgG, or TLR7/8 agonist (R848) as a positive control. We saw significant increases in IL-1β expression, on a per cell basis, as well as increases in the frequency of IL-1β expressing B cells when treated with agonistic CD40 and IL-21 (Fig 7A), mimicking the response observed in mouse splenic B cells stimulated with CD40 and IL-21 (Fig 2A–2C). Unlike in mouse B cells, stimulation of human B cells with CD40, IL-21, and anti-IgM/IgG resulted in significantly less IL-1β expression than in B cells stimulated with only CD40 and IL-21. Along with observations of lower IL-1β expression, we also observed that B cells stimulated with anti-IgM/IgG had significantly lower frequencies of live cells, therefore causing the decrease in IL-1β + B cells (S10C Fig). Seeing as there were comparable outcomes of IL-1β expression with *in vitro* stimulation of mouse and human B, we next examined IL-1β expression, *ex vivo*, in tonsillar B cells (S10D and S10E Fig). We observed GC B cells (CD19 + CD3-IgD-CD38+) had higher frequency of IL-1β+ cells and IL-1β intracellular expression than naïve B cells (Fig 7B), resembling our finding in mouse GC and naïve B cells (Fig 2E–2G). As IL-1β is expected to be produced by innate immune cells, we also examined IL-1β expression in monocyte and dendritic cell-like populations as a positive control of IL-1β expression in human tonsils. Both cell types expressed intracellular IL-1β (S10F Fig) and human B cells have previously been shown to produce IL-1β via NLRP3 and caspase-1 activity [43], therefore, we examined expression of active caspase-1. Similar to our observations in mouse GC B cells (Fig 3A and 3B), human GC B cells had a significantly higher quantity of active caspase-1 and higher percentage of cells with active caspase-1 (Fig 7C). To further confirm that human GC B cells are producing IL-1β, we observed significantly higher coexpression of IL-1β and active caspase-1 by human GC B cells (Fig 7D), supporting our hypothesis that in the human, GC B cells are the most likely IL-1β producers like their counterparts in mice.

In addition to the role of caspase-1 in IL-1β production in human GC B cells, we also wanted to determine if IL-1β production by human GC B cells is dependent on the NLRP3 inflammasome. We examined intracellular NLRP3 expression and unlike mouse GC B cells (Fig 4A), we did not observe differences in the frequency of NLRP3 expressing GC B cells and naïve B cells or in the amount of NLRP3 protein expressed between the two populations (Fig 7E). Although NLRP3 was not different between GC B cells and naïve B cells in human tonsils, GC B cells had significantly more coexpression of NLRP3 and active caspase-1 (Fig 7F). NLRP3 expression alone is not confirmation of NLRP3 inflammasome complex formation and activity but our observation of higher coexpression of active caspase-1 with both IL-1β and NLRP3 is a strong support for our hypothesis that similar to mouse GC B cells, human GC B cells produce IL-1β using an NLRP3 dependent mechanism.

We also examined naïve B cells in human PBMCs, as well as active B cells which would resemble the nGC population we examined in mice. In active B cells, we observe significantly more caspase-1 + cells than naïve B cells (S11A Fig). We further observed significantly higher intracellular IL-1β, both in the quantity of protein expression and frequency of IL-1β expressing cells, in active B cells than in naïve B cells in PBMCs (S11B Fig). The frequency of coexpression of caspase-1 and IL-1β was significantly higher in active B cells than in naïve B cells in PBMCs (S11C Fig), therefore suggesting the potential for active B cells in circulation to produce IL-1β. NLRP3 was not significantly different between active B cells and naïve B cells, in either frequency or protein quantity, in PBMCs (S11D Fig), however, coexpression of caspase-1 and NLRP3 was seen at significantly higher frequencies in active B cells of PBMCs than naïve (S11E Fig). Overall, our data shows that human B cells are a source of IL-1β, both in the palatine tonsils and in circulation, utilizing the NLRP3/Caspase-1 complex, like their murine counterparts.

## Discussion

The need for IL-1β within the GC has been suggested by reports of IL-1 signaling on TFH cells promoting TFH cell function and GC formation. The IL-1 axis within a GC has been confirmed by observation of IL-1 receptor signaling on TFH cells along with decoy and antagonist IL-1 receptors in TFR cell function. Predominantly using immunizations, this axis has been shown to play a significant role in GC regulation and antibody production [21,22]. Our work confirms the need for IL-1 signaling on TFH cells in influenza infection as we show that TFH cells, post influenza infection, express significantly more IL-1R1 and mice with IL-1R1 deficient T cells display significant reductions in numbers of TFH and GC B cell. While the importance of IL-1 signaling for T cell activation has been reported [27,44–46] and there may be other sources of IL-1β [27,47,48] outside the follicle, the source of IL-1β within the GC has not been previously examined. Cytokines within the GC act primarily in autocrine or paracrine signaling [49], therefore supporting the need for a local source of IL-1β for IL-1 signaling on TFH cells. As B cells are the most abundant population in the GC and have crosstalk with the TFH cells when they present antigen to them, we hypothesized the GC B cell to be the source of IL-1β. We demonstrate that GC B cells have significantly higher intracellular expression of IL-1β, compared to non-GC B cells, naïve B cells, and FDCs. As IL-1β activation is primarily executed by the zymogen caspase-1 we additionally examined active caspase-1 expression, again observing significantly higher expression of active caspase-1 in GC B cells than in naïve and nGC populations. To further support that GC B cells are producing IL-1β, not only expressing it, we examined coexpression of active caspase-1 and IL-1β, supporting that GC B cells, but not nGC and naïve B cells, produce IL-1β. Furthermore, using a B cell specific IL-1β KO mouse model we also show that B cell derived IL-1β is essential for optimal GC formation in influenza infection. Moreover, our observations of reduced T cell infiltration within the follicle and the GCs of mice with B cell specific IL-1β KO suggests that any source of IL-1β in the T cell zone is not sufficient for the TFH cell to traffic across the T-B border into the GC.

IL-1β production is a multi-step process, as it requires a priming step that promotes expression of pro-IL1β and an activation step that induces pro-IL1β cleavage into its mature form that can be released for signaling [50]. Activation of IL-1β requires formation of an inflammasome complex that will oligomerize with pro-caspase-1 and mediate its auto-activation, followed by active caspase-1 cleaving pro-IL1β into IL-1β [51]. Activation of active caspase-1 can also result in activation of gasdermin D (GSDMD), which is often associated with pyroptotic cell death [52]. While our examination of IL-1β was focused on live GC B cells, it is known that the highly proliferative GC compartment is simultaneously undergoing constant B cell death [53], therefore it would not be unlikely that B cells producing IL-1β will undergo cell death. Alternatively, studies have shown that not all cells secrete IL-1β by GSDMD-dependent mechanisms [54] and that cells can uncouple cytokine production and cell death post inflammasome activity [55], consequently leaving the questions of specific mechanisms for IL-1β secretion in B cells. Our findings support B cells to be a source of IL-1β within the GC, and regardless of cell death occurring post inflammasome activity, the need for IL-1β signaling on TFH cells can still be fulfilled.

Multiple inflammasomes can be involved in IL-1β activation, including NLRP3, NLRP1, NLRC4, AIM2, IFI16 and RIG-I [56,57], however because GC B cells are not directly infected by influenza infection, we examined NLRP3 and AIM2 as they are activated by stimuli likely to be present in GC B cells. NLRP3 does not directly detect microorganisms, but rather it can be activated by ROS, changes in ion flux, and extracellular ATP [57]. In B cells, both BCR stimulation and CD40 signaling can induce production of ROS [58]. ROS in B cells can promote B cell activation and proliferation [59]. BCR stimulation can promote an influx of calcium into the cytoplasm, which can induce NLRP3 inflammasome oligomerization. GC B cells deficient in endoplasmic reticulum (ER) calcium sensors that regulate the release of calcium from the ER show reduced GC B cell maintenance and decreased affinity maturation [60]. Additionally, B cells stimulated with CD40L and BCR ligation are able to secrete ATP, which in turn can activate the ion channel, P2X7R, allowing an efflux of potassium that can trigger NLRP3 inflammasome [61–63]. CD40 and BCR signaling were both observed to increase IL-1β expression following *in vitro* stimulation of B cells and these signaling mechanisms can also trigger production of stimuli that can lead to NLRP3 inflammasome activation. As NLRP3 can be activated by a variety of stimuli, many of which are present in GC B cells, we examined NLRP3 expression. We show GC B cell have significantly higher expression of NLRP3, in addition with higher coexpression of NLRP3-caspase-1 and NLRP3-IL1β. Furthermore, in B cell specific NLRP3 KO mice, we observed significant reduction in the number of GC B cells.

While we see a significant reduction of the GC in the absence of B cell NLRP3, we do not observe complete depletion of the GC. Therefore, we wondered if another mechanism of IL-1β could be compensating for the loss of NLRP3. Recurrent cell proliferation and mitochondrial remodeling that occurs in GC B cells [64], along with increase ROS and mitochondrial stress can result in the release of mitochondrial DNA into the cytoplasm [65]. As AIM2 expression can be upregulated with IFN-gamma signaling [66], and IFN-g + TFH cells peak at 10 days post influenza infection [67], we asked if AIM2 was expressed in GC B cells following influenza infection. Upon examination, we do observe upregulation of AIM2 expression in GC B cells following infection, however we do not see a significant impact of AIM2 activity on GCs and TFH cells. Perhaps as this is a redundant mechanism and NLRP3 appears to be the primary mechanism of IL-1β production in GC B cells we may not observe complete ablation of the GC unless B cells are deficient in both NLRP3 and AIM2.

Additionally, it is important to note that ablation of NLRP3 in B cells induces a more significant decrease in GC B cells than what is observed in B cell specific knockout of IL-1β. These observations bring into question other alternate roles of NLRP3 in GC B cells, including its effects on B cell selection, proliferation, and maturation within the GC. Inflammasome-independent roles of NLRP3 have been published, including its ability to translocate into the nucleus as act as a transcription factor in epithelial cells [68] and in CD4 T cells [69]. It has been shown that NLRP3 can act as a transcription factor for T_H_2 differentiation [69], therefore we cannot eliminate the possibility that NLRP3 may also act as a transcription factor in other lymphocytes, including B cells.

In human B cells we observe similar findings to mice, seeing as GC B cells from palatine tonsils express higher IL-1β than naïve B cells, as well as higher coexpression of active caspase-1 and NLRP3, suggesting that human GC B cells have the potential to produce IL-1β via the NLRP3 inflammasome. We also examined active B cells in human PBMCs, which also express IL-1β and coexpress active caspase-1 and NLRP3. As we find that active B cell in circulation could produce IL-1β, this prompts us to ask if B cell derived IL-1β is needed for mechanisms outside of the GC reaction. It would be important for future studies to examine the function of IL-1β production by B cells after memory cells and plasma cells exit the GC. In the context of a murine transplant model, B cell derived IL-1β is observed to be essential for T cell reconstitution [70]. Furthermore, atypical B cells are a source of IL-1β in a mouse model of systemic lupus erythematosus (SLE) [71], therefore we wonder if IL-1β observed in active circulating B cells could play a role in autoimmunity, be it in modulating T cell function or in actively taking part in autoimmune mediated pathology.

Collectively, our findings are significant as we have identified the vital source of IL-1β within the GC post influenza infection and identified the inflammasome that drives the production of this cytokine, which has conventionally been associated with innate cells. Our findings have unearthed a signaling pathway in the GCs post influenza infection which can promote TFH function and possibly the longevity of optimal GC function, which may be mirrored in human GC B cells. Therefore, we predict that these data will inform future studies on influenza vaccines, which aim to prolong optimal GC activity to achieve protection [42].

## Methods

### Ethics statement

Ethical approval for this study was obtained from the Institutional Review Board (IRB) at the Penn State College of Medicine (PSCOM). In this manuscript we use palatine tonsils and blood samples. The palatine tonsils are from routine surgical removal which is provided to us with no identifiers from the Pathology lab at PSCOM and the peripheral blood mononuclear cells are remnants from red blood cell phoresies filters from the Central Pennsylvania Blood Bank, which are provided with no identifiers. The PSCOM IRB protocol ID # is STUDY00018302. The PSCOM IRB classified this study “Non-Human Research”. Their reasoning was the following, **“**The Human Subjects Protection Office determined that the proposed activity, as described in the above referenced submission, does not meet the definition of human subject research as defined in 45 CFR 46.102(e) and/or (l). Institutional Review Board (IRB) review and approval is not required.**”**

The animal studies were approved by the Penn State College of Medicine Institutional Animal Care and Use Committee (IACUC) PHS Assurance Number: D16-00024 (A3045-01) USDA Registration Number: 23-R-0021. Allie Lab IACUC Approved protocol # PROTO202101794

### Mice and bone marrow transplantation for chimeric mice

Male and female C57BL/6J (CD45.2), B6.129S2-Ighmtm1Cgn/J (muMt-), B6.129S6-Nlrp3tm1Bhk/J (Nlrp3-/-), B6.129P2-Tcrbtm1Mom/J (Tcrβ-), B6.129S7-Il1r1tm1Imx/J (Il1r1^-/-^), B6.129P2-Aim2Gt(CSG445)Byg/J (Aim2^-/-^) were purchased from The Jackson Laboratory (Bar Harbor, ME) and Il-1β^-/-^ mice (developed by David Chaplin (UAB, Birmingham AL) and kindly provided by Jenny Ting (UNC Chapel Hill, NC)) and bred at the Pennsylvania State University College of Medicine vivarium. muMt^-^ or Tcrβ^-^ recipient mice were lethally irradiate with a total dose of 850 rad. 80/20 B cell specific bone marrow chimeras were made with bone marrow from muMt^-^: Knockout (Nlrp3^-/-^, Il1β^-/-^, or Aim2^-/-^), muMt^-^: Wild-type, Tcrβ^-^: Knockout (Il1r1^-/-^) and Tcrβ^-^: Wild-type prepared at a ratio of 80:20. Recipient mice received a bone marrow transplant of 5–10 $\times 10^{6}$ fresh cells intravenous injection. Recipient mice were used in experiments after 8–10 weeks allowing for complete reconstitution of the hematopoietic compartment.

### Infections

Influenza H1N1 A/PR8/34 (A/PR8) strain was used for primary. A/PR8 was administered intranasally at 15,000 viral foci units (VFU).

### Tissue processing and flow staining

Mediastinal lymph nodes were isolated and mechanically disrupted to prepare a single cell suspension. Fluorochrome-conjugated antibodies were titrated in staining wash buffer (SWB; 2% fetal bovine serum in PBS) to stain cells. 4% formalin fixation was used for surface stains and the Foxp3/Transcription factor buffer set (eBiosciences) was used for intracellular staining. The Far-Red Fluorescent FLICA 660 Caspase-1 (YVAD) assay kit (Immunochemistry Technologies, Davis, CA) was used to stain for active caspase-1 following manufacturers’ specifications. For flow staining for coexpression of IL-1β or NLRP3 and active caspase-1, samples were incubated with FLICA, followed by a short 15min cell fixation in 1% paraformaldehyde and a 10min permeabilization with 0.5% Tween-20. The BD FACSSymphony (BD Biosciences, Franklin Lakes, NJ) was used to perform flow cytometry and a BD Aria SORP high performance cell sorter (BD Biosciences, Franklin Lakes, NJ) was used for sorting experiments, at the Penn State College of Medicine Flow Cytometry core (RRID:SCR_021134).

### Human tonsil and PBMCs processing

Low density mononuclear cells were isolated from tonsils. Tonsils were mechanically disrupted and passed through a 70um strainer to obtain a single cell suspension. Peripheral blood mononuclear cells were isolated from blood drained from filter blood bags. Using a density gradient, the single cell suspension was separated by centrifugation with lymphocyte separation medium (Corning, Corning, NY). Tonsil cells and PBMCs were stained for flow cytometry.

### B cell isolation and *in vitro* cell stimulation

B cells were enriched from spleen or lymph node tissues using immunomagnetic negative selection kit (STEMCELL Technologies, Cambridge, MA). B cells seeded at 2$\times 10^{5}$ cells/well in 96-well round bottom plates. Mouse cells were stimulated with 10u/mL mouse IL-2 (MilliporeSigma, Rockville, MD), 5ng/mL recombinant BAFF (BioLegend, San Diego, CA),10ug/mL anti-mouse CD40 (BioXcell, Lebanon,NH), 10ng/mL recombinant IL-21 (Peprotech, Cranbury, NJ), 10ug/mL Goat anti-mouse IgM (Jackson ImmunoResearch, West Grove, PA) or 5ug/mL R848/Resiquimod (MilliporeSigma, Rockville, MD). Human cells were stimulated with 5ug/mL goat anti-human IgG (Jackson ImmunoResearch, West Grove, PA), 5ug/mL goat anti-human IgM (Jackson ImmunoResearch, West Grove, PA), 5ug/mL R848 (InvivoGen, San Diego, CA), 50U/mL recombinant IL-2, 10ng/mL recombinant IL-21 (Peprotech, Cranbury, NJ), 20ng/mL recombinant IFNg (R&D Systems, Inc, Minneapolis, MN), 10ng/mL recombinant BAFF, and/or 10ug/mL agonistic anti-CD40 (BioXcell, Lebanon,NH). Cells were stimulated for 48hr and were stained for flow cytometry.

### Immunofluorescence microscopy

#### Tissue.

Isolated lymph nodes were embedded in optimal cutting temperature (O.C.T.) compound (Sakura Finetek, Torrance, CA). Lymph nodes were submerged in 2-Methylbutane brought to temperature in liquid nitrogen to snap freeze tissues. Tissues were cut into 6μm sections using a cryostat (Hacker Instruments and Industries, Winnsboro, SC). Sections were mounted on Colorfrost Plus Microscope Slides (Fisher Scientific, Waltham, MA) and acetones was used as a fixative. Tissue sections were stained with GL7-FITC, CD4-PE, IgD-APC (BD Biosciences, Franklin Lakes, NJ).

#### Colocalization.

Single cell suspension from lymph nodes from A/PR8 infected mice were pooled and sorted for naïve B cells (CD19 + IgD+) and GC B cells (CD19 + GL7+). 50,000 naïve or GC B cells were mounted on Colorfrost Plus Microscope Slides (Fisher Scientific) using a Thermo Shandon Cytospin 3 Centrifuge (Marshall Scientific, Hampton, NH). 4% paraformaldehyde was used as a fixative and 0.5% Tween-20 was used for permeabilization. Cells were stained with GL7-AF488 or IgD-AF488 (BioLegend, San Diego, CA) and biotin anti-mouse IL-1β (BioLegend, San Diego, CA) and polyclonal Caspase-1 antibody (Invitrogen, Carlsbad, CA). Secondary antibody AF555-conjugated streptavidin (Invitrogen, Carlsbad, CA) was used for IL-1β detection and AF647-conjugated donkey anti-Rabbit IgG (BioLegend, San Diego, CA) was used for caspase-1 detection. DAPI (MilliporeSigma, Rockville, MD) at 5μg/mL was used for nuclear staining. Cells were imaged on the Leica SP8 laser scanning confocal microscope (Leica Microsystems, Buffalo Grove, IL, USA) available at the Penn State College of Medicine advanced light microscopy core (RRID:SCR_022526). Data analysis for colocalization imaging was performed using the Imaris Software (Version 10) (Oxford Instruments, Switzerland). The “spot co-localization” feature was used to identify co-localizing spots of IL-1β and caspase-1. Spots that were ≤0.4μm from each other were determined to be co-localized [72].

### Statistics

Data analysis was performed on GraphPad Prism software (Version 10, GraphPad Software, Inc.). Data were reported as mean ± standard deviation (SD). Comparisons between 2 groups were analyzed using Welch’s t-test, unpaired t-test, or Mann-Whitney test. Comparisons between 3 or more groups were performed using Ordinary one-way analysis of variance (ANOVA) with Turkey’s multiple comparison test or Holm-Šídák test. Comparisons with a P-value <0.05 were considered to be statistically significant.

## Supporting information

S1 Fig(A) Gating strategy for identification of TFH cells examined in Fig 1.Bcl-6 expression in (**B**) nTFH compared to TFH cells and (**C**) compared between CD44^-^, CD44^int^, and CD44^hi^ CD4 T cells. (**D**) IL1R1 + gating in nTFH and TFH populations to determine IL1R1 + frequencies used to calculate IL1R1 + TFH cells numbers in Fig 1C. (**E**) IL1R1 expression quantified by MFI and frequency between nTFH, CXCR5-PD-1+ and TFH cells. (**F**) CD44 expression of TFH and nTFH cells and (**G**) gating strategy for identification of TFH cells and nTFH cells from CD4 T cell population expressing different levels of CD44, and IL1R1 expression based on CD44 expression. Data are representative of 2 experiments with 4–5 mice (**B-C**) and 2 experiments with 5 mice (**E**) and graphs show individual points and mean± SD. *p < 0.05, **p < 0.01, ***p < 0.001, and ****p < 0.0001.(TIFF)

S2 Fig (A) Gating strategy for live, singlet lymphocytes. (**B**) A dump gate was used to identify cells that are CD8a- Gr-1- F4/80- Ter-119-. From the dump- gate, TFH cells were gated as CD19-CD4 + CD25-CXCR5 + PD-1 + cells and GC B cells were gated as CD19 + CD95 + GL7 + .
(TIFF)

S3 Fig (A) gating strategy used to identify live, singlet lymphocytes form culture B cells. (**B**) IL-1β + B cell numbers were quantified in different stimulation groups used in Fig 2A and 2C. Splenic B cells were enriched from (**C**) naïve or (**D**) A/PR8 infected mice at 15 dpi and stimulated with a combination of rBAFF, rIL2, R848, anti-CD40, rIL21 to examine the number of B cells expressing IL-1β. Data are representative of 3 experiments with technical triplicates per stimulation group (**B-D**) and graphs show individual points and mean±SD. *p < 0.05, **p < 0.01, ***p < 0.001, and ****p < 0.0001.
(TIFF)

S4 Fig(A) Gating strategy of live, singlet lymphocytes used to identify (B) naïve, GC, and nGC B cells population.(**C**) Comparison of IL-1β expression in each respective population with their respective FMO (red) and the gating used to identify frequencies of IL-1β+ cells and determine MFI of IL-1β IC expression. (**D**) Blc-6 intracellular staining quantified and compared between naïve, nGC, and GC B cells. (**E**) IC IL-1β expression was examined in GL7-, GL7^Dim^, and GL7^Hi^ B cell populations and IL-1β was quantified by (**F**) MFI and frequency. Data are representative of 2 experiments with 4–5 mice (**D**) 3 experiments with 3 mice (**E**) and graphs show individual points and mean± SD. *p < 0.05, **p < 0.01, ***p < 0.001, and ****p < 0.0001.(TIFF)

S5 Fig(A) Gating strategy of live, singlet lymphocytes used to identify (B) naïve, GC, and nGC B cells population used for analysis in Fig 4.(**C**) Follicular dendritic cells (FDCs) were gated as CD19-CD4-CD21 + CD35+ and active B cells (act. B cells) were gated as CD19 + IgD- to compare intracellular IL-1β expression and active caspase-1 expression. (**D**) Dark zone (DZ) and light zone (LZ) frequencies from GC B cells were quantified and these population were examined in Fig 3E. (**D**) Data is representative of 2 experiments with 4 mice examined at 10dpi. Graphs show individual points and mean±SD. *p < 0.05, **p < 0.01, ***p < 0.001, and ****p < 0.0001.(TIFF)

S6 Fig Gating strategy used to sort (A) Naïve B cells which were surface stained for IgD and (B) GC B cells surfaced stained for GL7. Cells identified as IgD or GL7 positive during confocal imaging were used for IL-1β and caspase-1 colocalization in Fig 3F–3H. Naive (**C**) and GC (**D**) B cells stained from IL-1β and caspase-1 colocalization representative of the 20 cells per group imaged.
(TIFF)

S7 Fig (A) Gating strategy of live, singlet lymphocytes used to identify (B) naïve, GC, and nGC B cells population. (TIFF)

S8 Fig (A) Gating strategy for live, singlet lymphocytes. (**B**) A dump gate was used to identify cells that are CD8a- Gr-1- F4/80- Ter-119-. From the dump- gate, TFH cells were gated as CD19-CD4 + CD25-CXCR5 + PD-1 + cells and GC B cells were gated as CD19 + CD95 + GL7 + . This gating was used to determine GC and TFH cells population in B-WT and B-IL1β^-/-^ mice (Fig 5). Immunofluorescent imaging was used to examine GC B cells (GL7, green), naïve B cells (IgD, blue) marking the B cell follicle, and helper T cells (CD4, red) (**C**) to examine GCs and CD4 + T cell infiltration into the B cell follicle and the GC. (**D**) Gating strategy for live, singlet lymphocytes. (**E**) A dump gate was used to identify cells that are CD8a- Gr-1- F4/80- Ter-119-. From the dump- gate, TFH cells were gated as CD19-CD4 + CD25-CXCR5 + PD-1 + cells and GC B cells were gated as CD19 + CD95 + GL7 + . This gating was used to determine GC and TFH cells population in B-WT and B-NLRP3^-/-^ mice (Fig 5).
(TIFF)

S9 Fig (A) Gating strategy for AIM2 + cells following staining with an unconjugated rabbit anti-mouse AIM2 antibody followed by an APC-conjugated goat anti-rabbit secondary antibody. (**B**) Gating strategy for live, singlet lymphocytes. (**C**) A dump gate was used to identify cells that are CD8a- Gr-1- F4/80- Ter-119-. From the dump- gate, TFH cells were gated as CD19-CD4 + CD25-CXCR5 + PD-1 + cells and GC B cells were gated as CD19 + CD95 + GL7 + . This gating was used to determine GC and TFH cells population in B-WT, and B-AIM2^-/-^ mice (Fig 6).
(TIFF)

S10 Fig (A) Gating strategy for live, singlet lymphocytes from cultured B cells isolated from human tonsils. (**B**) CD19 + B cells were gated to determine IC IL-1β expression in stimulated tonsillar B cells. (**C**) Gating for live cell population in each *in vitro* stimulation group. (**D**) Gating strategy for live, singlet lymphocytes in human tonsils and PBMCs. (**E**) Gating strategy to identify naïve (CD19 + CD3-CD38-IgD+) and GC (tonsils) and activated (PBMCs) (CD19 + CD3-CD38 + IgD-) B cells. Each population was further analyzed for NLRP3, IL-1β, and active caspase-1 expression. (**F**) CD3- cells were further dated into CD68 + HLA-DR + CD11b- cells and CD68-HLA-DR + CD11b- to examined IC IL-1β.
(TIFF)

S11 Fig Naïve and active B cells (Act. B) from human PBMCs, were compared for (A) active caspase-1, (B) IC IL-1β, and (C) coexpression of IL-1β and active caspase-1. Naïve and act. B cells were also examined for (**D**) intracellular NLRP3 expression and (**E**) coexpression of NLRP3 and active caspase-1. (**A-E**) Data are representative of 2 experiments with 4–5 patient samples per experiment. *p < 0.05, **p < 0.01, ***p < 0.001, and ****p < 0.0001.
(TIFF)

S1 Table List of antibodies used. (XLSX)

S2 Table Statistical analysis of Figs 1–7. (XLSX)

S3 Table Statistical analysis of supplementary figures. (XLSX)
