# Supplementary figures and images for: Germinal Center B cells provide essential IL-1β signals to TFH cells via canonical NLRP3 inflammasome activity post influenza infection
Source: PLoS Pathog. 2025 Aug 18;21(8):e1013404. doi: 10.1371/journal.ppat.1013404 (PMC12373279; doi:10.1371/journal.ppat.1013404)

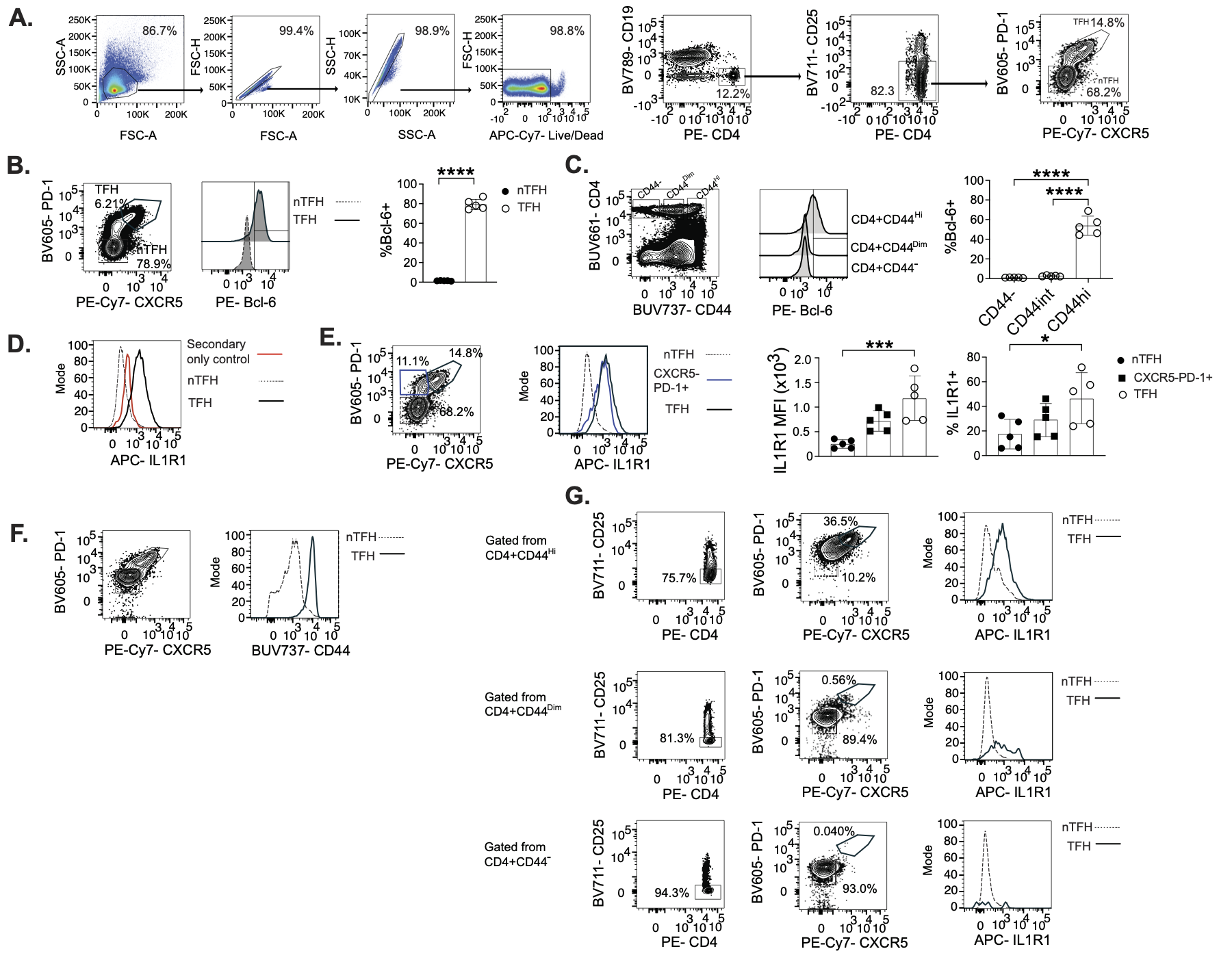

Supplement: S1 Fig — Bcl-6 expression in (B) nTFH compared to TFH cells and (C) compared between CD44-, CD44int, and CD44hi CD4 T cells. (D) IL1R1 + gating in nTFH and TFH populations to determine IL1R1 + frequencies used to calculate IL1R1 + TFH cells numbers in Fig 1C. (E) IL1R1 expression quantified by MFI and frequency between nTFH, CXCR5-PD-1+ and TFH cells. (F) CD44 expression of TFH and nTFH cells and (G) gating strategy for identification of TFH cells and nTFH cells from CD4 T cell population expressing different levels of CD44, and IL1R1 expression based on CD44 expression. Data are representative of 2 experiments with 4–5 mice (B-C) and 2 experiments with 5 mice (E) and graphs show individual points and mean± SD. *p < 0.05, **p < 0.01, ***p < 0.001, and ****p < 0.0001. (TIFF) [file ppat.1013404.s001.tiff]

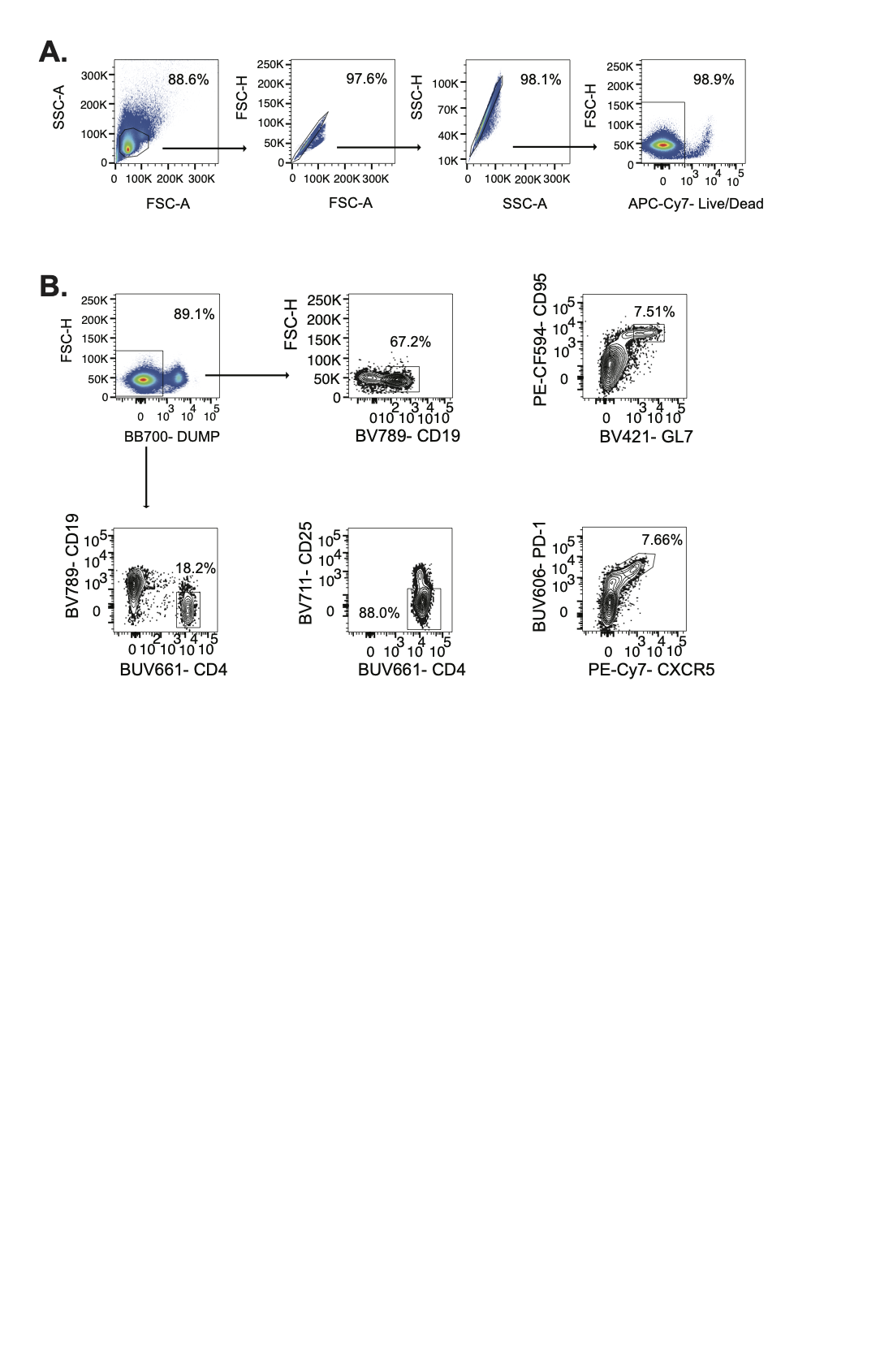

Supplement: S2 Fig — (B) A dump gate was used to identify cells that are CD8a- Gr-1- F4/80- Ter-119-. From the dump- gate, TFH cells were gated as CD19-CD4 + CD25-CXCR5 + PD-1 + cells and GC B cells were gated as CD19 + CD95 + GL7 + . (TIFF) [file ppat.1013404.s002.tiff]

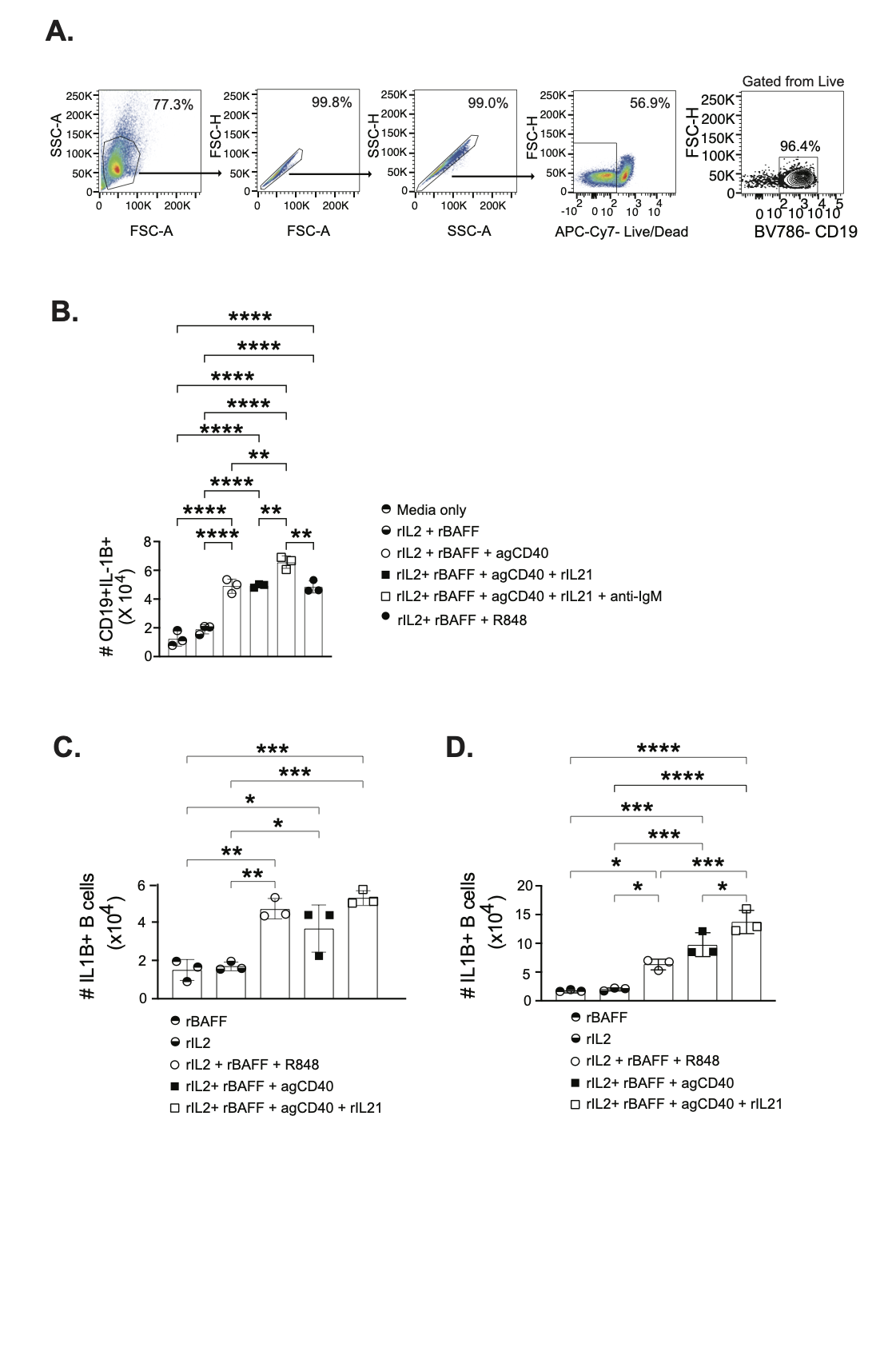

Supplement: S3 Fig — (B) IL-1β + B cell numbers were quantified in different stimulation groups used in Fig 2A and 2C. Splenic B cells were enriched from (C) naïve or (D) A/PR8 infected mice at 15 dpi and stimulated with a combination of rBAFF, rIL2, R848, anti-CD40, rIL21 to examine the number of B cells expressing IL-1β. Data are representative of 3 experiments with technical triplicates per stimulation group (B-D) and graphs show individual points and mean±SD. *p < 0.05, **p < 0.01, ***p < 0.001, and ****p < 0.0001. (TIFF) [file ppat.1013404.s003.tiff]

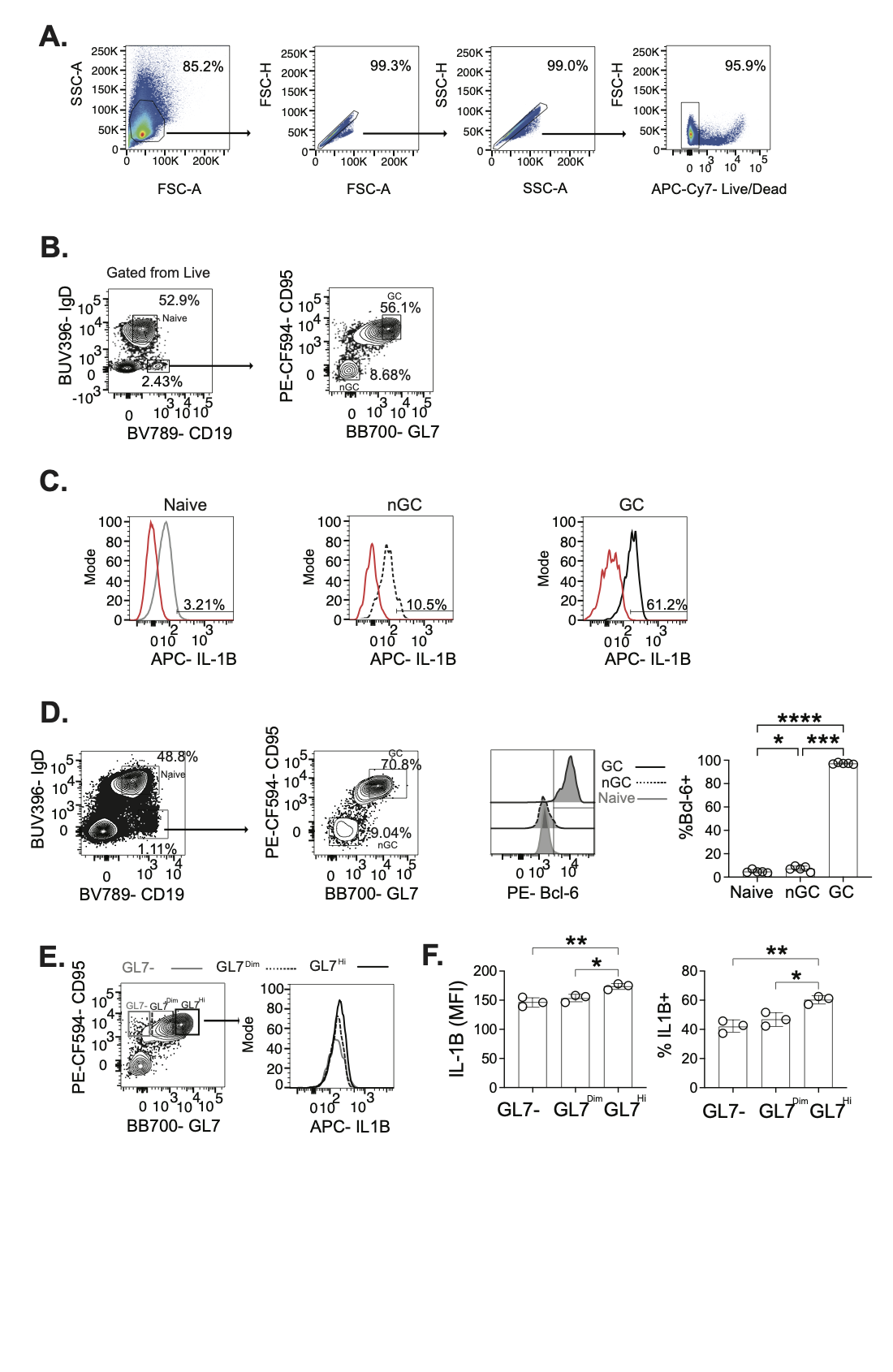

Supplement: S4 Fig — (C) Comparison of IL-1β expression in each respective population with their respective FMO (red) and the gating used to identify frequencies of IL-1β+ cells and determine MFI of IL-1β IC expression. (D) Blc-6 intracellular staining quantified and compared between naïve, nGC, and GC B cells. (E) IC IL-1β expression was examined in GL7-, GL7Dim, and GL7Hi B cell populations and IL-1β was quantified by (F) MFI and frequency. Data are representative of 2 experiments with 4–5 mice (D) 3 experiments with 3 mice (E) and graphs show individual points and mean± SD. *p < 0.05, **p < 0.01, ***p < 0.001, and ****p < 0.0001. (TIFF) [file ppat.1013404.s004.tiff]

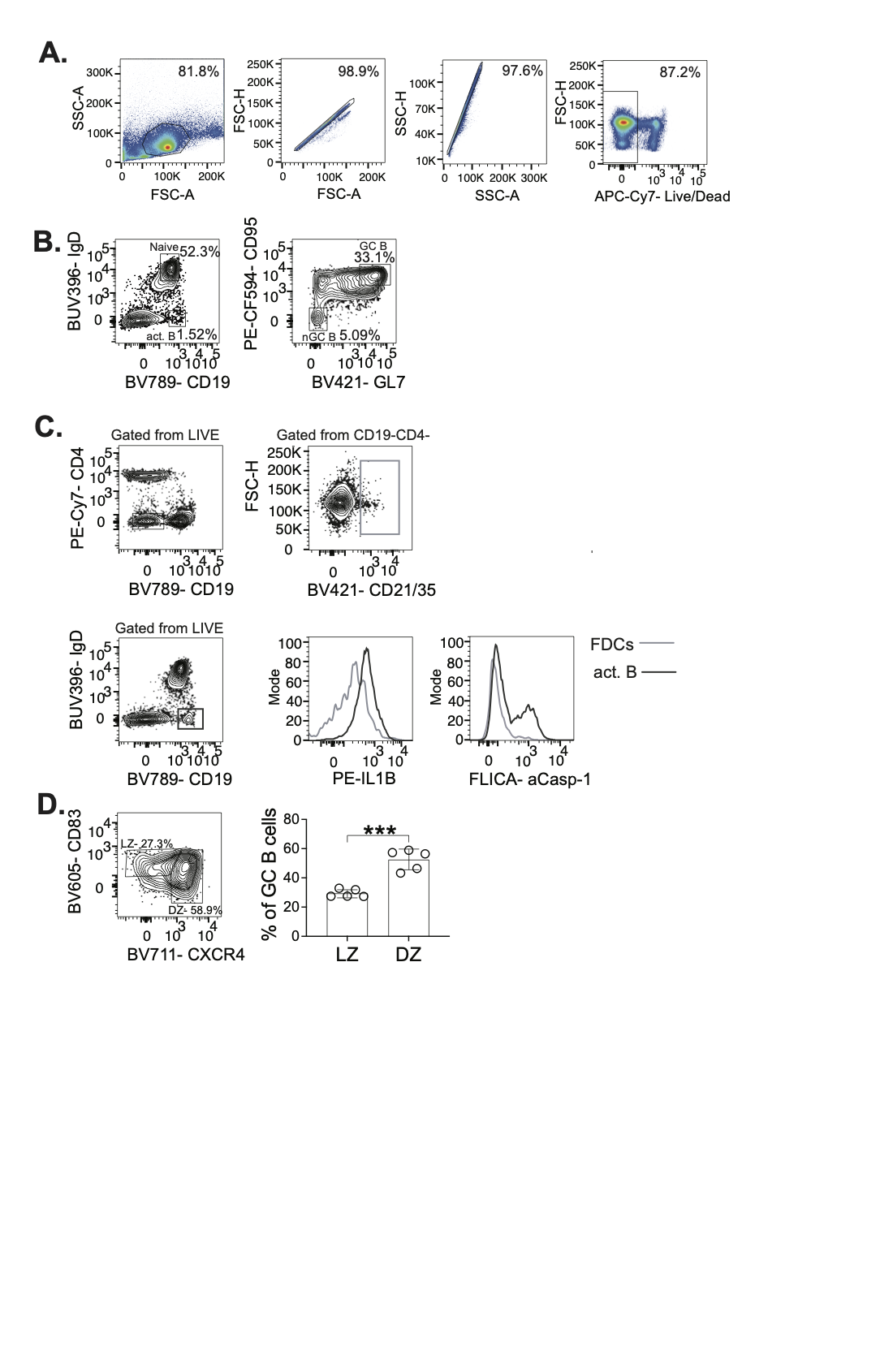

Supplement: S5 Fig — (C) Follicular dendritic cells (FDCs) were gated as CD19-CD4-CD21 + CD35+ and active B cells (act. B cells) were gated as CD19 + IgD- to compare intracellular IL-1β expression and active caspase-1 expression. (D) Dark zone (DZ) and light zone (LZ) frequencies from GC B cells were quantified and these population were examined in Fig 3E. (D) Data is representative of 2 experiments with 4 mice examined at 10dpi. Graphs show individual points and mean±SD. *p < 0.05, **p < 0.01, ***p < 0.001, and ****p < 0.0001. (TIFF) [file ppat.1013404.s005.tiff]

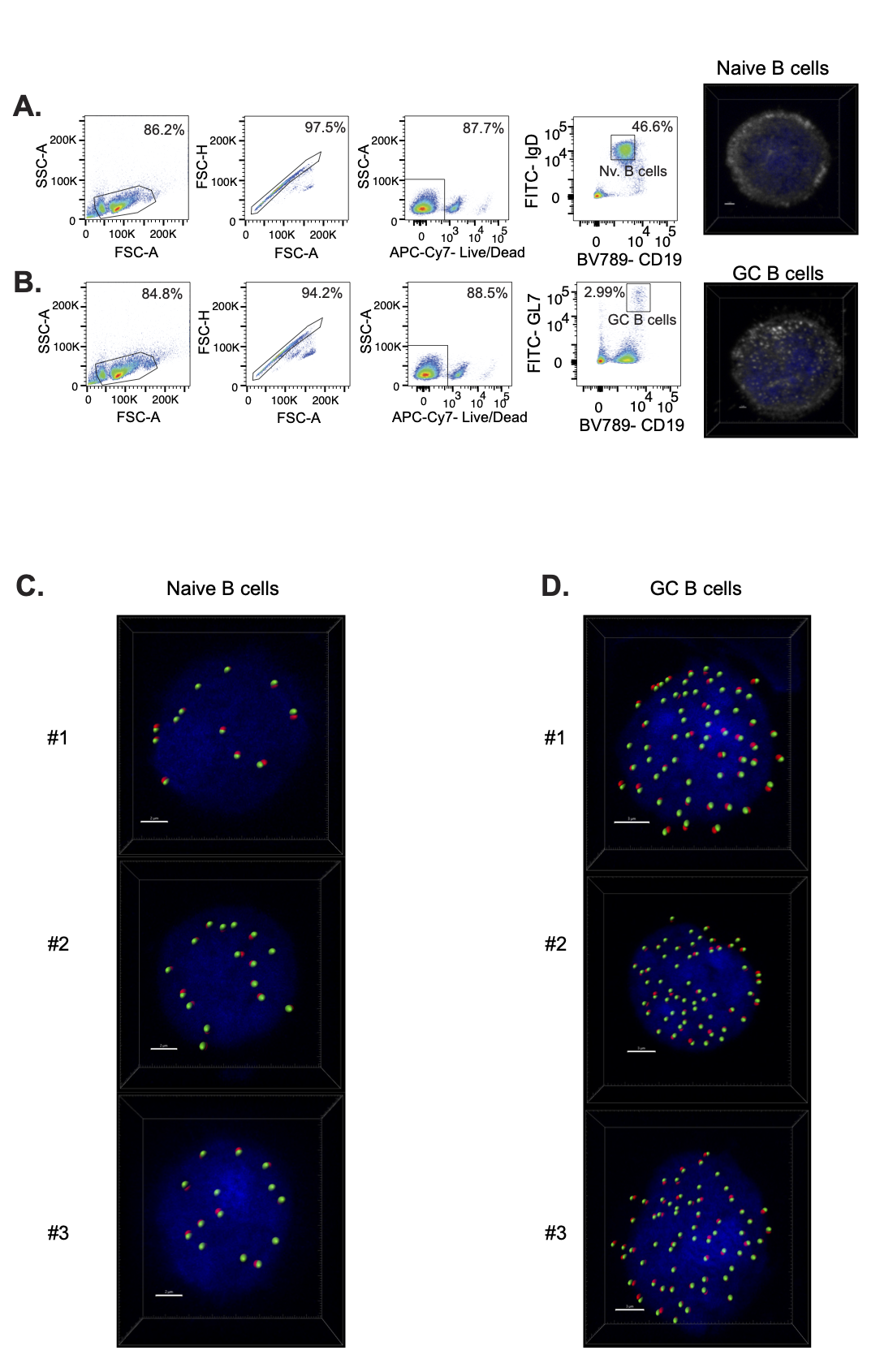

Supplement: S6 Fig — Cells identified as IgD or GL7 positive during confocal imaging were used for IL-1β and caspase-1 colocalization in Fig 3F–3H. Naive (C) and GC (D) B cells stained from IL-1β and caspase-1 colocalization representative of the 20 cells per group imaged. (TIFF) [file ppat.1013404.s006.tiff]

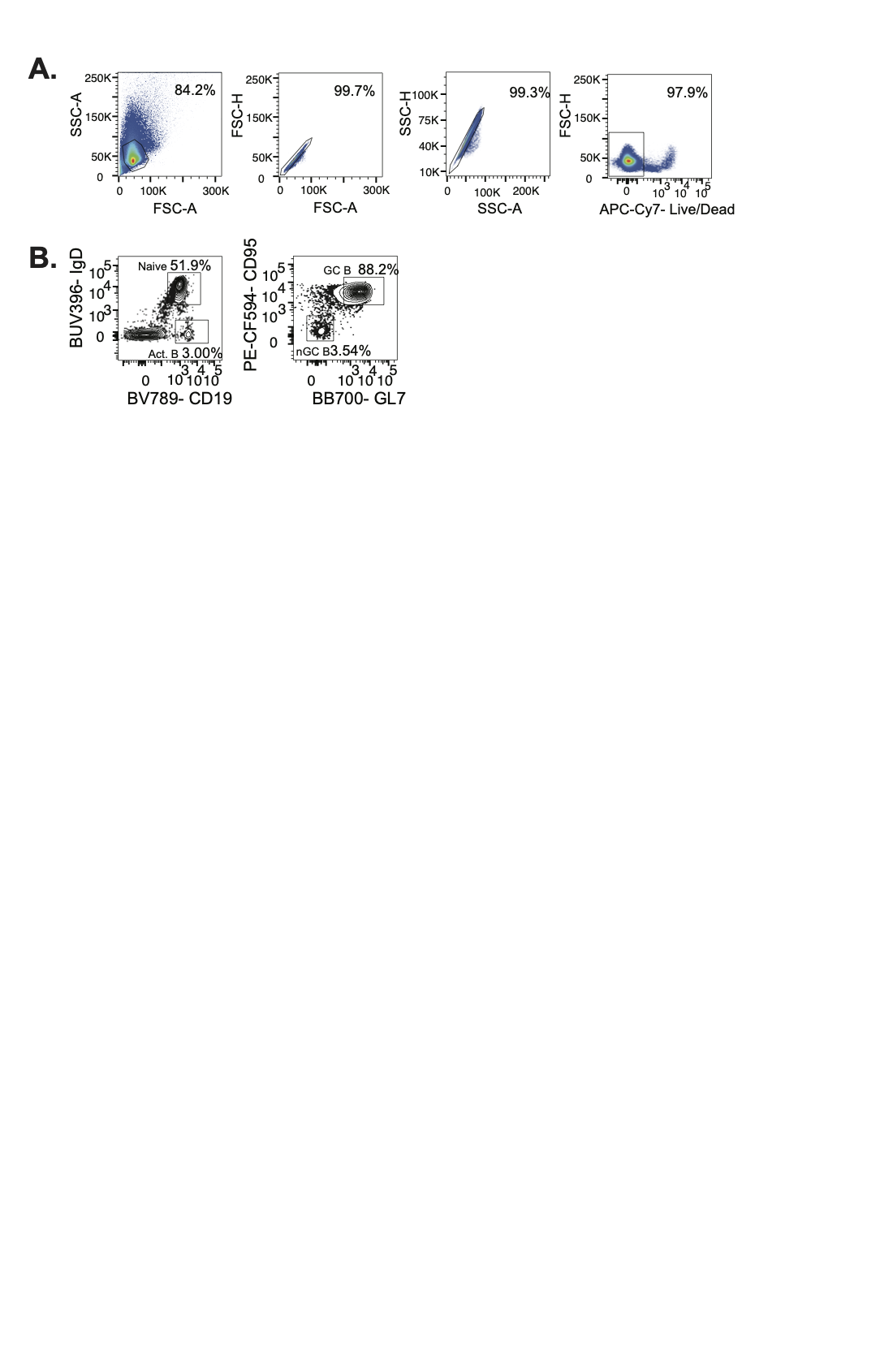

Supplement: S7 Fig — (TIFF) [file ppat.1013404.s007.tiff]

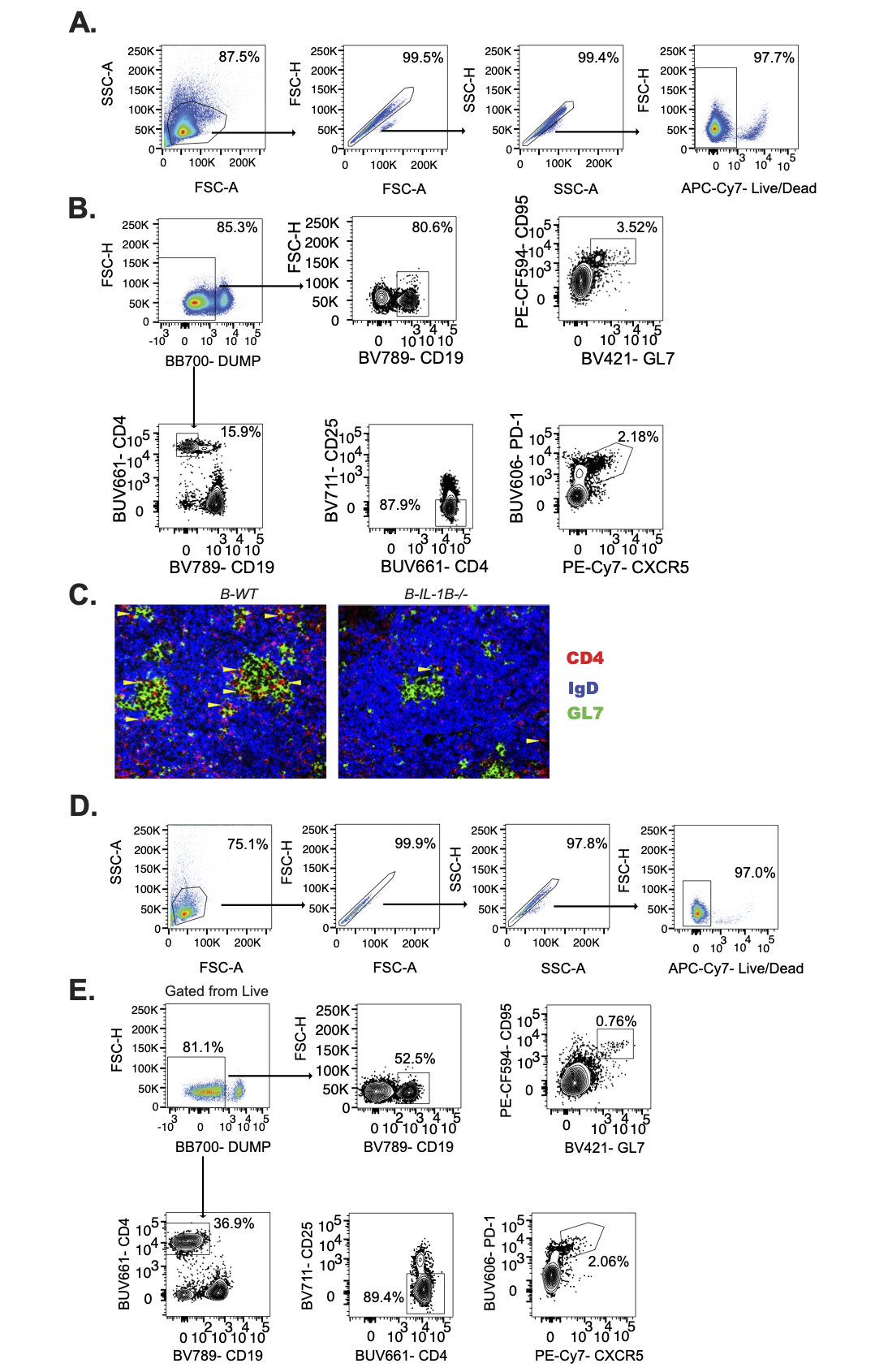

Supplement: S8 Fig — (B) A dump gate was used to identify cells that are CD8a- Gr-1- F4/80- Ter-119-. From the dump- gate, TFH cells were gated as CD19-CD4 + CD25-CXCR5 + PD-1 + cells and GC B cells were gated as CD19 + CD95 + GL7 + . This gating was used to determine GC and TFH cells population in B-WT and B-IL1β-/- mice (Fig 5). Immunofluorescent imaging was used to examine GC B cells (GL7, green), naïve B cells (IgD, blue) marking the B cell follicle, and helper T cells (CD4, red) (C) to examine GCs and CD4 + T cell infiltration into the B cell follicle and the GC. (D) Gating strategy for live, singlet lymphocytes. (E) A dump gate was used to identify cells that are CD8a- Gr-1- F4/80- Ter-119-. From the dump- gate, TFH cells were gated as CD19-CD4 + CD25-CXCR5 + PD-1 + cells and GC B cells were gated as CD19 + CD95 + GL7 + . This gating was used to determine GC and TFH cells population in B-WT and B-NLRP3-/- mice (Fig 5). (TIFF) [file ppat.1013404.s008.tiff]

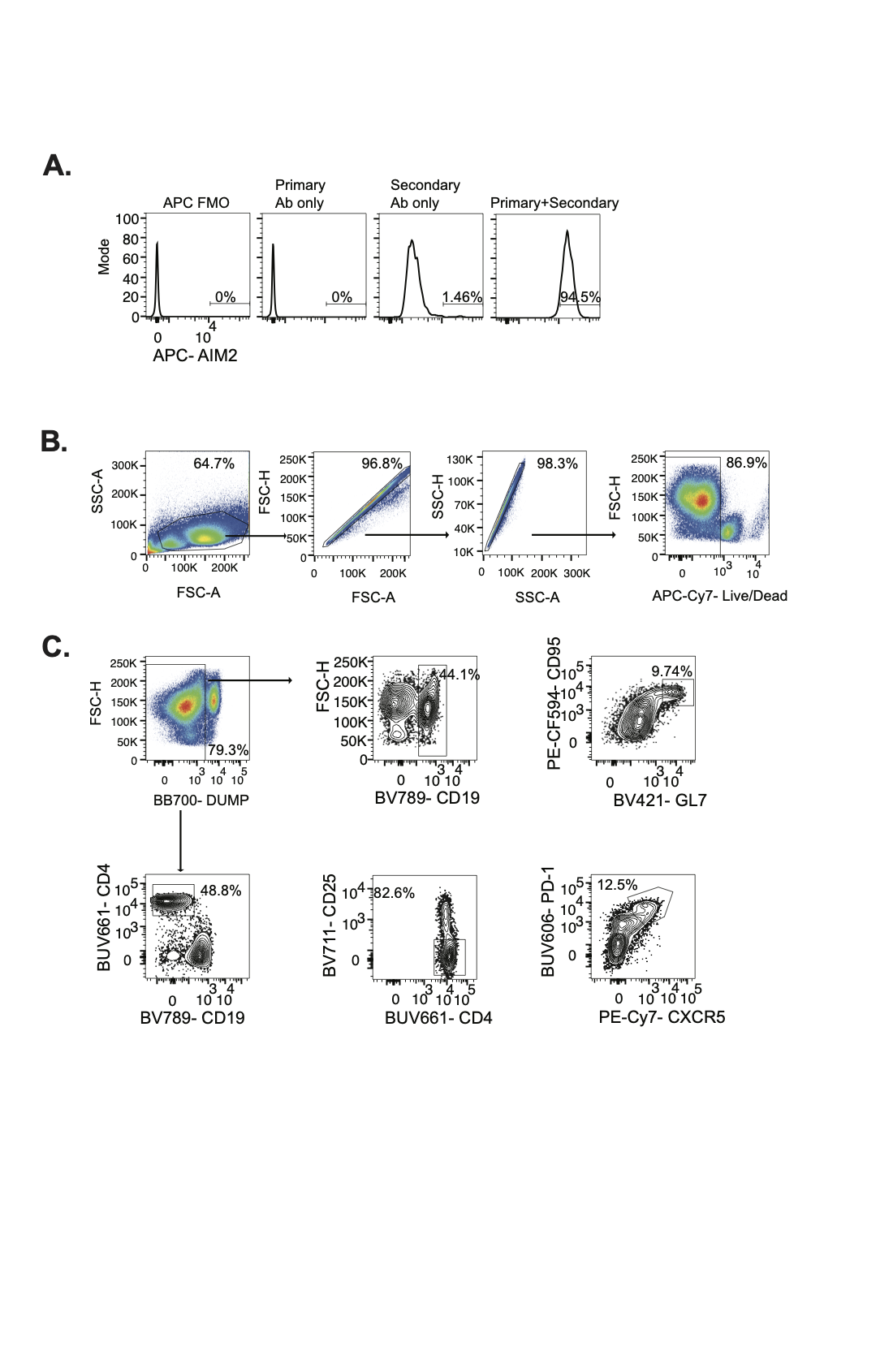

Supplement: S9 Fig — (B) Gating strategy for live, singlet lymphocytes. (C) A dump gate was used to identify cells that are CD8a- Gr-1- F4/80- Ter-119-. From the dump- gate, TFH cells were gated as CD19-CD4 + CD25-CXCR5 + PD-1 + cells and GC B cells were gated as CD19 + CD95 + GL7 + . This gating was used to determine GC and TFH cells population in B-WT, and B-AIM2-/- mice (Fig 6). (TIFF) [file ppat.1013404.s009.tiff]

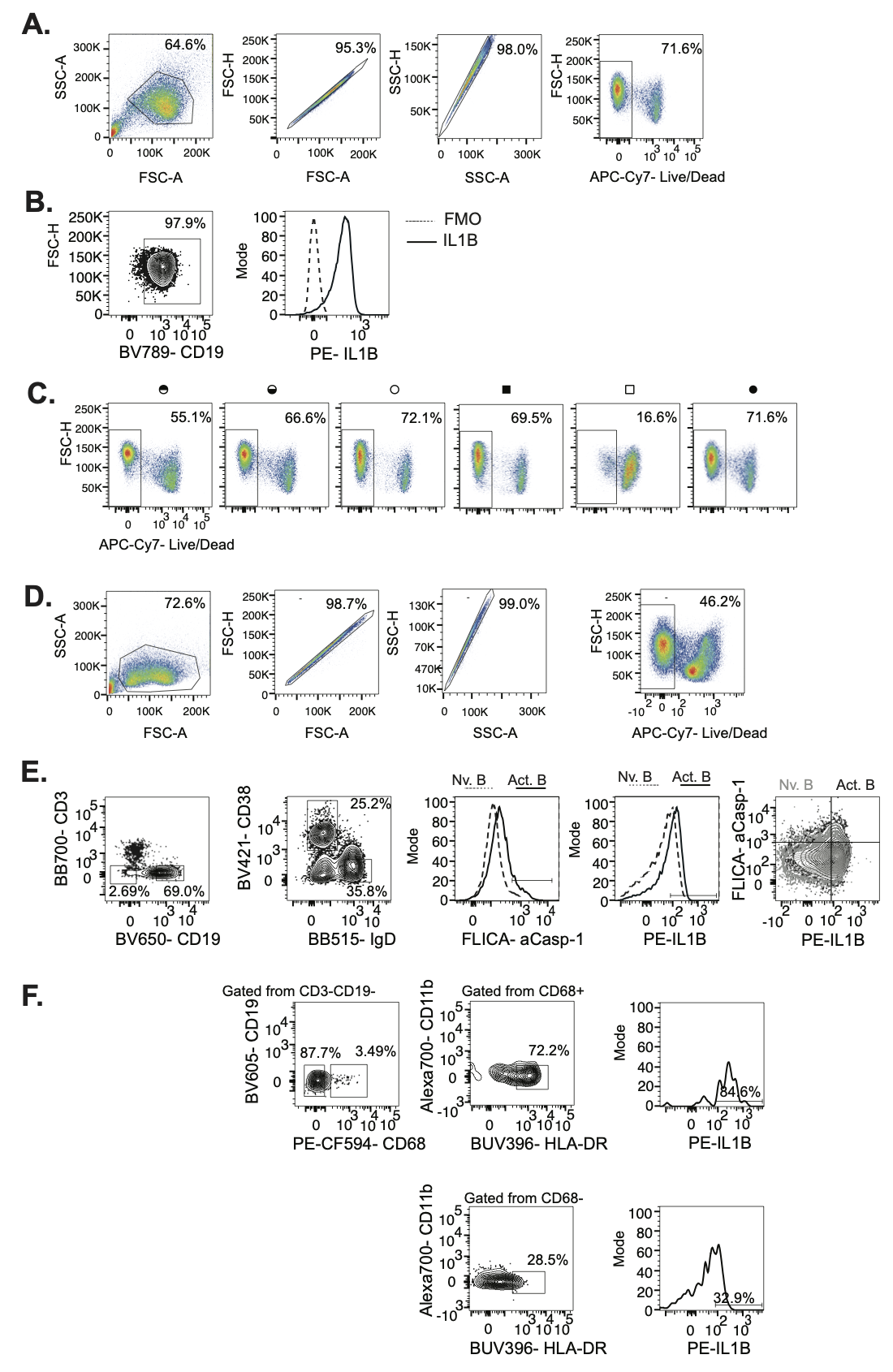

Supplement: S10 Fig — (B) CD19 + B cells were gated to determine IC IL-1β expression in stimulated tonsillar B cells. (C) Gating for live cell population in each in vitro stimulation group. (D) Gating strategy for live, singlet lymphocytes in human tonsils and PBMCs. (E) Gating strategy to identify naïve (CD19 + CD3-CD38-IgD+) and GC (tonsils) and activated (PBMCs) (CD19 + CD3-CD38 + IgD-) B cells. Each population was further analyzed for NLRP3, IL-1β, and active caspase-1 expression. (F) CD3- cells were further dated into CD68 + HLA-DR + CD11b- cells and CD68-HLA-DR + CD11b- to examined IC IL-1β. (TIFF) [file ppat.1013404.s010.tiff]

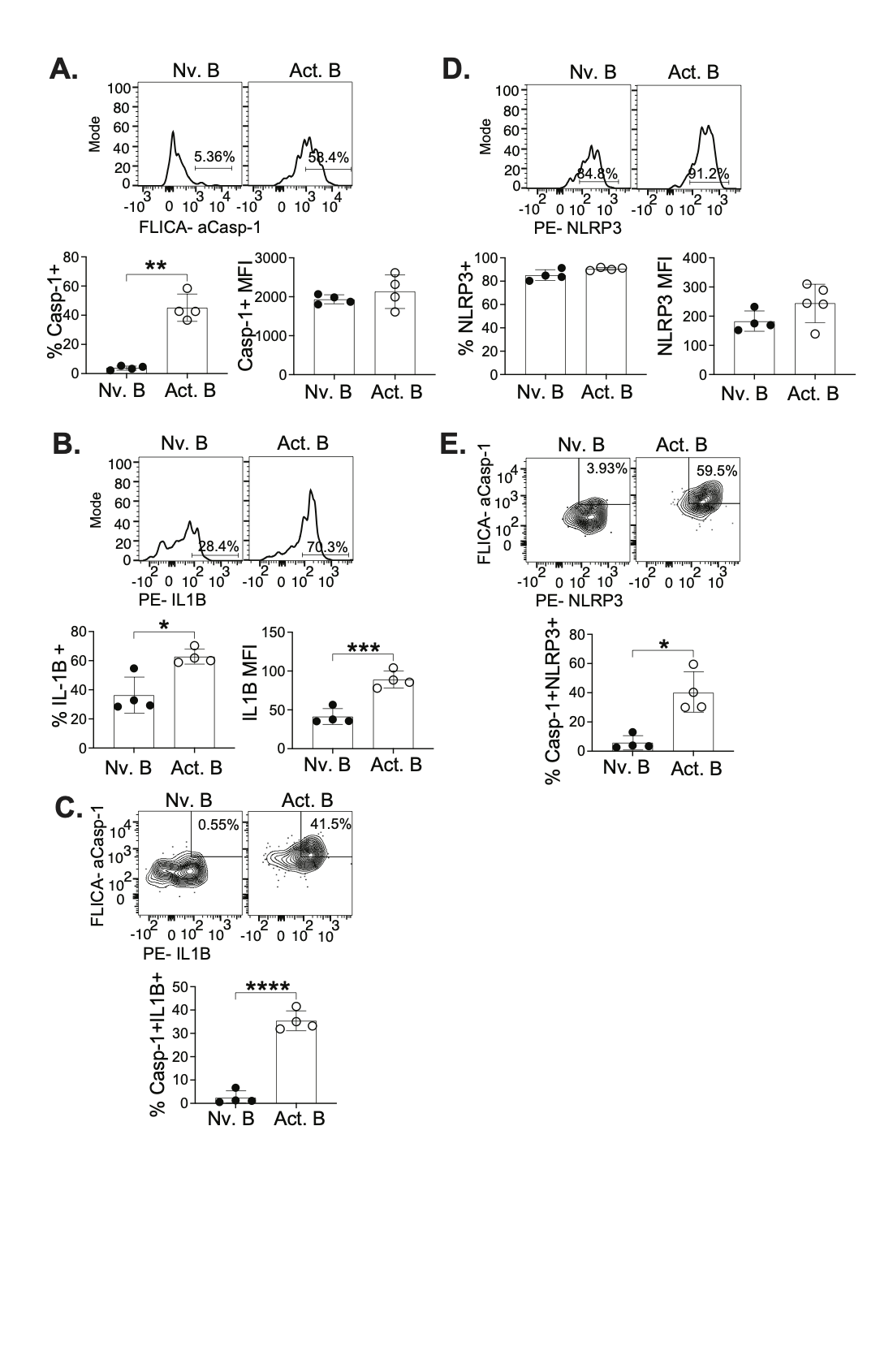

Supplement: S11 Fig — Naïve and act. B cells were also examined for (D) intracellular NLRP3 expression and (E) coexpression of NLRP3 and active caspase-1. (A-E) Data are representative of 2 experiments with 4–5 patient samples per experiment. *p < 0.05, **p < 0.01, ***p < 0.001, and ****p < 0.0001. (TIFF) [file ppat.1013404.s011.tiff]
